# Fundamental Role of Methylenetetrahydrofolate Reductase 677 C → T Genotype and Flavin Compounds in Biochemical Phenotypes for Schizophrenia and Schizoaffective Psychosis

**DOI:** 10.3389/fpsyt.2016.00172

**Published:** 2016-11-09

**Authors:** Stephanie Fryar-Williams

**Affiliations:** ^1^Youth in Mind Research Institute, Norwood, SA, Australia; ^2^The Queen Elizabeth Hospital, Woodville, SA, Australia; ^3^Basil Hetzel Institute for Translational Health Research, Woodville, SA, Australia

**Keywords:** psychosis, MTHFR 677C → T polymorphisms, riboflavin, copper, schizophrenia

## Abstract

The Mental Health Biomarker Project (2010–2016) explored variables for psychosis in schizophrenia and schizoaffective disorder. Blood samples from 67, highly characterized symptomatic cases and 67 gender and age matched control participants were analyzed for methyl tetrahydrofolate reductase (MTHFR) 677C → T gene variants and for vitamin B6, B12 and D, folate, unbound copper, zinc cofactors for enzymes in the methylation cycle, and related catecholamine pathways. Urine samples were analyzed for indole-catecholamines, their metabolites, and oxidative-stress marker, hydroxylpyrolline-2-one (HPL). Rating scales were Brief Psychiatric Rating Scale, Positive and Negative Syndrome Scale, Global Assessment of Function scale, Clinical Global Impression (CGI) score, and Social and Occupational Functioning Assessment Scale (SOFAS). Analysis used Spearman’s correlates, receiver operating characteristics and structural equation modeling (SEM). The correlative pattern of variables in the overall participant sample strongly implicated monoamine oxidase (MAO) enzyme inactivity so the significant role of MAO’s cofactor flavin adenine nucleotide and its precursor flavin adenine mononucleotide (FMN) within the biochemical pathways was investigated and confirmed as 71% on SEM of the total sample. Splitting the data sets for MTHFR 677C → T polymorphism variants coding for the MTHFR enzyme, discovered that biochemistry variables relating to the wild-type enzyme differed markedly in pattern from those coded by the homozygous variant and that the hereozygous-variant pattern resembled the wild-type-coded pattern. The MTHFR 677C → T-wild and -heterozygous gene variants have a pattern of depleted vitamin cofactors characteristic of flavin insufficiency with under-methylation and severe oxidative stress. The second homozygous MTHFR 677TT pattern related to elevated copper:zinc ratio and a vitamin pattern related to flavin sufficiency and risk of over-methylation. The two gene variants and their different biochemical phenotypes govern findings in relationship to case-identification, illness severity, duration of illness, and functional disability in schizophrenia and schizoaffective psychosis, and establish a basis for trials of gene-guided precision treatment for the management of psychosis.

## Introduction

The Mental Health Biomarker Project (MHBP, 2010–2013 and ongoing) was designed to discover and explore biomarkers capable of discriminating between those participants with and without a functional psychosis condition, as mainly represented by schizophrenia and schizoaffective disorder. Candidate markers had already been explored by pilot study (Section S1 in Supplementary Material) with promising results. These and other candidate markers were selected for assay and results examined by receiver operating characteristic (ROC) curve analysis in order to discover their biomarker status. Then, the most outstanding biomarkers were incorporated into a model for case-detection and prediction for screening purposes (1).

As part of the biomarker exploration component of this project, biomarkers of the functional psychosis model were examined for their predictive translational relationships. As a result, biochemistry-nutrition domain biomarkers were found to exert subtle, yet fundamental cumulative effects on neurotransmitter synthesis and metabolism (2). Such biomarkers possessed significant interactions with each other in domains representing elevated catecholamines, oxidative stress, and visual and auditory processing abnormalities. Schizophrenia and schizoaffective psychosis was, therefore, confirmed to be a composite, multi-domain entity where nutrition-related biomarkers exert the strongest overall predictive influence on all other biomarker domains. This fundamental biochemistry domain was, therefore, considered to be an important entity requiring further exploration.

The MHBP found particular biomarkers for low folate, vitamin B6, and vitamin D (1) that have known epidemiological links with schizophrenia (3). Hydroxyhemopyrroline-2-one (HPL) was also found to be a biomarker and this is a theoretical indicator of oxidative stress and disturbed porphyrin synthesis with heme-degredation in schizophrenia (4). A further significant finding from the MHBP was that noradrenaline (NA), adrenaline (AD), and their metabolic product methoxy-hydroxymandelic acid (MHMA), when configured as NA/MHMA and AD/MHMA, to represent activity of their metabolizing enzyme monoamine oxidase (MAO), formed highly significant biomarkers for schizophrenia and schizoaffective psychosis. This finding highlighted the need to analyze variables and biomarkers relating to MAO activity and its flavin cofactor (FAD) in relationship to catecholamine turnover and caseness for functional psychosis (1, 2).

**Figure 1:**
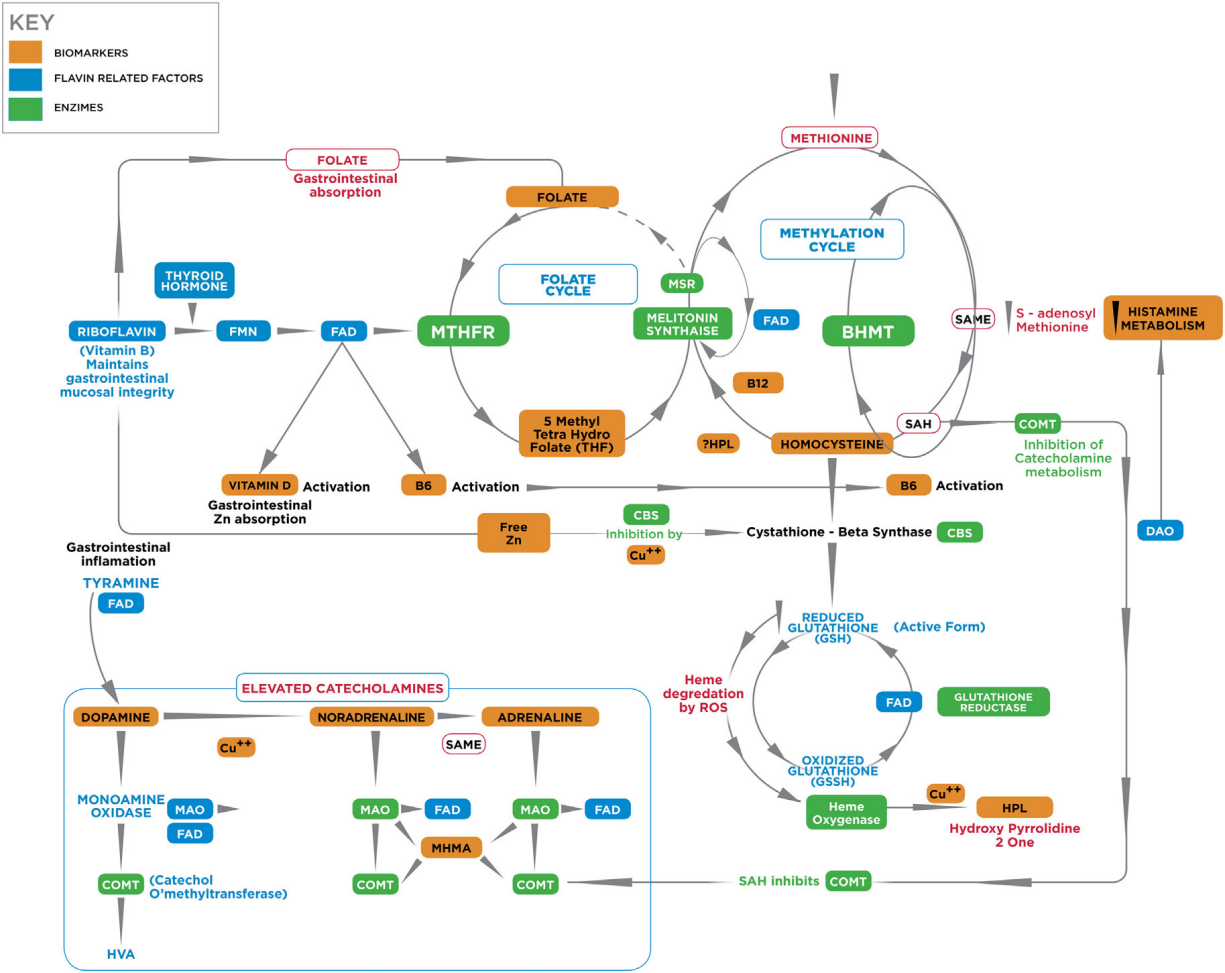
**A diagrammatic outline of the biochemical territory covered in the MHBP and presented in this manuscript**. Enzymes: BHMT, betaine homocysteine methyltrasferase; COMT, catechol-o-methyl-transferase; CBS, cystathione beta synthase, MAT, methionine adenosyltransferase, MTHFR, methylenetetrahydrofolate reductase, SAMe, S-denosylmethionine, MT, methyltransferase, SAHH, S-adenosylhomocysteine-hydrolase, MSR, methionine sulfoxide reductase, MS, methionine synthase. Vitamin cofactors: vitamin B6 (pyridoxine), vitamin B12 (cobalamin), vitamin C, folic acid, 5 methyl tetrahydrofolate. Mineral enzyme cofactors: free (unbound) copper (Cu), zinc. Intermediate substrates: BH4, tetrahydrobiopterin BH2 – dihydrobiopterin; DMG, Dimethylglycine; DOPAL, dihydroxyphenylacetaldehyde; DOPAC, dihydroxyphenylacetic acid; DOPEGAL, dihydroxyphenylglycolaldehyde; DOMA, dihydroxymandelic acid; DHPG, dihydroxyphenylglycal; DOPA, dihydroxyphenylalanine; FAD, flavin adenine dinucleotide, 5HIAA, 5-hydroxyindolacetic acid, HVA, homovanillic acid, MAO, monoamine oxidase; MHMA, 3-methoxy-4-hydroxymandelic acid; MHPG, 4-hydroxy-3-methoxyphenylglycol; SAH, *S*-adenosylhomocysteine; TMG, trimethylglycine, VMA, vanillylmandelic acid. HPL, urinary hydroxyhemopyrroline-2-one.

Figure 1 depicts known interactive biochemical pathway relationships derived from research literature sources. In a bicyclic process referred to as one-carbon metabolism, the folate cycle is coupled to the methionine (methylation) cycle through the generation of 5-methyl-THF (MTHF) by the flavin-dependent enzyme methyl tetrahydrofolate reductase (MTHFR). Protein for this enzyme is coded by the MTHFR 677 C → T gene where Cytosine may be replaced by thymidine at the 677 position (5). Through metabolism of homocysteine (HCY) at the methionine synthase (MS) junction point between the folate and methionine cycles, MTHF donates a carbon to HCY to generate methionine, which in turn generates *S*-adenosylmethionine (SAMe). As a major methyl donor in cells, SAMe contributes to histone, DNA and RNA methylation and, therefore, to epigenetic regulation of gene expression (6, 7). SAMe is also an important cofactor for the second step of catechol-o-methyl transferase (COMT) metabolism of catecholamines as well as a cofactor for conversion of NA to AD (8). At the bottom of the methylation cycle, the vitamin B6-dependent transsulfuration pathway is connected to the methionine cycle through HCY, leading to the generation of cysteine and eventually glutathione, one of the major redox-regulating agents in cells (9). In addition, interactions between free copper, vitamin B6, catecholamine synthesis, and glutathione synthesis pathways have been outlined in literature reviews (10, 11).

## Materials and Methods

A full outline of Section “Materials and Methods” for this study can be found in previously published papers (1, 2), and a brief summary is given below for the interest of readers.

This study was approved by the Queen Elizabeth Hospital Research Ethics Committee (No: 2009139) and protocols and methods conformed to that committee’s regulatory standards. The author reports no conflict of interest at the time of undertaking this research or writing this paper. A provisional patent application was filed in September 2016. Participants were assessed at the Queen Elizabeth Hospital and the Basil Hetzel Institute for Translational Health at Woodville, South Australia and two satellite psychiatric clinics in the Western Adelaide community catchment area (for further information, see Section “Investigation of MTHFR 677TT Gene Status in Relationship to Key Variables Using a Split Data Set”). Though the study was designed to be completed within 3 years, with data collection between May 2010 and December 2014, the study has been extended to assess a further catchment area and is still ongoing.

Recruitment of patients with schizophrenia and schizoaffective disorder and controls lacking these disorders was from multi-ethnic backgrounds in an age-range between 18 and 60 years. The aim of recruitment was to impose sufficient exclusion criteria to minimize confounding variables and strip psychosis in the case sample as far as possible down to its bare functional form (S2). In this way, potential confounding effects of substance abuse, organic causes, and medication were minimized and candidate markers that have strong discrimination and case-detection efficacy for functional psychosis could be exposed and be matched for age and sex with control participants.

Antipsychotic medication remained stable during the assessment period and DSM IV-R criteria (12) case diagnoses were made by trained staff and checked by consensus opinion and DSM IV-R symptom-checklist. Patients and controls were rated for clinical and subclinical symptoms, respectively, and had biological samples taken prior to auditory and visual processing assessments. Control participants were randomly letter and phone-recruited from participants in the North West Adelaide catchment area after age and sex – stratified based upon patient recruitment data. Similar exclusion criteria as for patient recruitment were imposed and though no control participants possessed a diagnosis of mental illness, they were rated for subclinical symptoms in a similar manner to case participants. Further information on this process can be found in Sections S2, S3 in Supplementary Material.

Participants were assessed in a real-world setting, by psychiatrically trained assessors who were not blind to their participants’ status but were blind to their laboratory and sensory processing status. Demographic data and information regarding risk predictors for schizophrenia or schizoaffective disorder (such as presence or absence of a family history of schizophrenia, depression or mania, developmental disorder history or learning disorder history, history of ear infection, premorbid subclinical (non-concussed) head injury, hospitalization-frequency, disability-pension-status), was collected prior to rating scale assessments. Outcome measure ratings utilized the Clinical Global Impressions Scale (CGI) for severity, Global Assessment of Function (GAF), Social and Occupational Functioning Assessment Scale (SOFAS), and symptoms were rated using the Brief Psychiatric Rating Scale (BPRS) amalgamated with the Positive and Negative Syndrome for schizophrenia (PANSS) (S3). A symptom intensity rating (SIR, rated 1–7) was derived for each symptom and taken as an additional measure of clinical severity.

Candidate biochemical markers were selected based on pilot study results and for citations already-outlined in the Section “Introduction.” Standardized collection methods, protocols, and citations are documented in Sections S4 and S5 in Supplementary Material. Blood was assayed by commercial laboratories for vitamin B6, vitamin B12, red cell folate, plasma HCY, serum copper, serum ceruloplasmin, red cell zinc, serum histamine, methyltetrahydrofolate reductase (MTHFR 677 C → T) gene polymorphism, and vitamin D. Urine assays were for levels of creatinine, dopamine (DA), NA, AD, and two of their metabolites [homovanillic acid (HVA), MHMA] as well as the serotonin metabolite 5-hydroxyindoleacetic acid (5-HIAA) and the oxidative stress biomarker HPL.

### Background Analysis and Results

Sample characteristic analysis, including characteristics related to medication and risk factors had previously been examined using XLSTAT (13) and STATA software (14). ROC curve analysis (15, 16) used both XLSTAT and STATA software in order to discover which candidate markers met criteria for biomarkers of schizophrenia and schizoaffective disorder.

Due to implementation of the multiple exclusion criteria and an eligible participant consent rate of only 1 in 4, recruitment was slow and unpredictable, however, the process did result in a highly characterized group of patients becoming enrolled in the study and final data analysis was based on data from 67 cases and 67 control participants. The biochemical biomarker results are summarized in S6. A full report of results from this study can also be found in Fryar-Williams and Strobel (1, 2). Details of catchment area characteristics, recruitment process outcomes, participant medications and data imputation can be found in S7. On Spearman’s correlation analysis, the nutritional-biochemical domain ROC held highly significant correlates with outcome measures for functional psychosis severity (CGI and SIR), disability (GAF, disability pension and SOFAS) and hospital admission rate (at 95% level of significance) (1) (Table 1).

**Table 1:** **Nutritional biochemistry domain, elevated catecholamine and oxidative stress domain ROCs, with respect to functional measures of disability and severity**.

Domain ROCs	Case versus control	SOFAS ROC	GAF ROC	CGI ROC	Hospital admission rate	Disability pension requirement	Symptom intensity rating (SIR)
High catecholamines	0.598	0.591	0.562	0.591	0.583	0.460	0.4677
Nutrition-related biochemistry	0.458	0.415	0.415	0.415	0.403	0.309	0.404
Oxidative stress (HPL/creatinine)	0.339	0.312	0.315	0.312	0.421	0.296	0.327

*All rho values are significant at 95% level of confidence. For rho ≥ 0.350, P ≤ 0.0001*.

*SOFAS, social and occupational function scale. GAF, Global Assessment of Function Scale. CGI, Clinical Global Index (of Severity). Hospital admission rate as determined by number of admissions divided by duration of illness and Disability Pension receipt, as an index of cost burden*.

### Further Statistical Analysis

**Figure 2:**
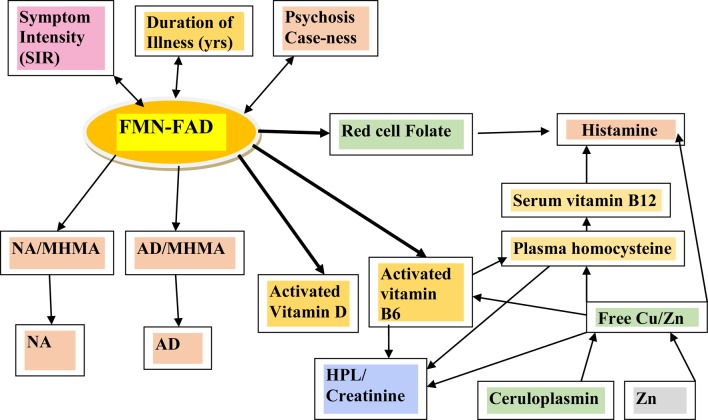
**Variables examined by SEM analysis in the context of their relationship within the biochemical pathways of interest**.

Spearman’s correlation analysis (17) was first performed to explore and understand relationships between candidate marker levels and compound ROC variables in relationship to all other biochemical variables (Tables 2–4). Structural equation modeling (SEM) was secondly performed using STATA software (14) to examine the predicted role of Riboflavin-related flavin compounds FMN and FAD as intermediary variables in the biochemical pathways of interest (Figure 2; Table 5). SEM analysis employs a system of simultaneous equations to determine to what degree the overall sample data support the linkage of any putative independent variable to other dependent variables in the data set and in the pathway of the model. Goodness of fit coefficient (R2) of the putative model is then calculated as a measure of percentage variation in the data. Finally, Spearman’s correlation analysis (17) was conducted on three data-sets, after data had been split three ways according to MTHFR 677 C → T genotype (wild type, heterozygous polymorphism type, or homozygous polymorphism type). The data-sets so obtained were examined in relationship to other variables as well as caseness and functional measures for severity (CGI), symptom intensity (SIR), disability (GAF), and social and occupational function (SOFAS) (Tables 6 and 7). Dynamic understandings derived from these three different levels of data analysis were then collated and aligned in order to obtain overall perspective of the dynamics at work within the biochemistry of schizophrenia and schizoaffective psychosis.

**Table 2:** **Total sample correlation analysis for biochemical variables**.

Candidate marker variables		VitD	RC folate	B6	Se B12	Free Cu/Zn		Vit D	RC folate	B6	Se B12	Free Cu/Zn	MTHFR homozygous
Caseness	Correlation coefficient	−0.262	−0.268	−0.240	0.113	0.141	AD	−0.248	−0.210	−0.187	−0.019	−0.011	
	Sig. (two-tailed)	**0.002**	**0.002**	**0.006**	0.195	0.103		**0.004**	**0.015**	**0.034**	0.825	0.901	
	*N*	132	133	129	134	134		131	132	128	133	133	
SIR index	Correlation coefficient	−0.209	−0.209	−0.124	0.192	0.082	AD/MHMA	−0.186	−0.186	−0.118	−0.030	−0.050	
	Sig. (two-tailed)	**0.016**	**0.016**	0.162	**0.026**	0.345		**0.036**	**0.035**	0.189	0.737	0.571	
	*N*	132	133	129	134	134		128	129	125	130	130	
GAF	Correlation coefficient	0.234	0.335	0.335	0.230	−0.061	HPL/CREAT	−0.137	−0.033	−0.066	0.104	−0.030	−0.181
	Sig. (two-tailed)	0.009	0.000	0.000	0.165	0.499		0.120	0.704	0.456	0.232	0.731	**0.037**
	*N*	122	123	123	124	124		131	132	128	133	133	133
CGI	Correlation coefficient	−0.276	−0.275	−0.205	0.140	0.112	Plasma HCY	−0.036	−0.213	−0.188	−0.049	−0.052	0.224
	Sig. (two-tailed)	**0.002**	**0.002**	**0.025**	0.121	0.216		0.683	**0.015**	**0.033**	**0.581**	**0.551**	**0.009**
	*N*	122	123	119	124	124		130	131	128	132	132	134
SOFAS	Correlation coefficient	0.216	0.286	0.259	−0.120	−0.072	Free Cu/Zn	0.008	0.062	−0.125	−0.060	1.000	
	Sig. (two-tailed)	**0.017**	**0.001**	**0.004**	0.184	0.424		0.928	0.478	0.157	0.493		
	*N*	122	123	119	124	124		132	133	129	134	134	
DA	Correlation coefficient	−0.236	−0.212	−0.102	−0.057	−0.097	B6	0.120	0.355	1.00	0.301	−0.125	
	Sig. (two-tailed)	**0.007**	**0.015**	0.254	0.516	0.266		0.178	**0.000**		**0.001**	0.157	
	*N*	131	132	128	133	133		127	128	129	129	129	
DA/HVA	Correlation coefficient	−0.107	−0.180	−0.099	−0.176	0.039	Se B12	0.079	0.112	0.301	1.00	−0.060	
	Sig. (two-tailed)	0.223	**0.039**	**0.264**	**0.043**	0.655		0.371	0.198	**0.001**		0.493	
	*N*	131	132	128	133	133		132	133	129	134	134	
NA	Correlation coefficient	−0.298	−0.224	−0.247	−0.031	0.005	RC folate	0.257	1.000	0.355	0.112	0.062	
	Sig. (two-tailed)	**0.001**	**0.010**	**0.005**	0.723	0.957		**0.003**		**0.000**	0.198	0.478	
	*N*	131	132	128	133	133		131	133	128	133	133	
NA/MHMA	Correlation coefficient	−0.198	−0.167	−0.203	−0.035	0.002	Vit D	1.00	0.257	0.120	0.079	0.008	
	Sig. (two-tailed)	**0.025**	**0.059**	**0.023**	0.695	0.984			**0.003**	0.178	0.371	0.928	
	*N*	128	129	125	130	130		132	131	127	132	132	

**Table 3:** **Total sample correlation matrix results for ROC biochemistry variables in relationship to each other**.

ROC biochemistry variable correlates		Low VitD ROC AUC = 0.65	Low RC folate ROC AUC = 0.654	Low B6 ROC AUC = 0.638	High Se B12 ROC AUC = 0.565 (Sig 80%)	High Free Cu:Zn × 100 ROC AUC = 0.611	MTHFR_hom	High HIST ROC AUC = 0.576
Caseness	Correlation coefficient	0.268	0.31	0.3	0.146	0.225	−0.034	0.164
	Sig. (two-tailed)	**0.002**	**0**	**0.001**	0.093	0.009	0.7	**0.058**
	*N*	132	133	129	134	133	134	134
SIR Index	Correlation coefficient	0.243	0.258	0.162	0.236	0.158	−0.003	0.147
	Sig. (two-tailed)	**0.005**	**0.003**	0.066	**0.006**	0.069	0.975	0.091
	*N*	132	133	129	134	133	134	134
GAF	Correlation coefficient	−0.243	−0.377	−0.251	−0.122	−0.109	0.023	−0.074
	Sig. (two-tailed)	**0.007**	**0**	**0.006**	0.176	0.23	0.801	0.411
	*N*	122	123	119	124	123	124	124
CGI	Correlation coefficient	0.302	0.309	0.231	0.143	0.145	−0.021	0.113
	Sig. (two-tailed)	**0.001**	**0.001**	**0.012**	0.112	0.109	0.815	0.212
	*N*	122	123	119	124	123	124	124
SOFAS	Correlation coefficient	−0.243	−0.33	−0.286	−0.13	−0.124	0.022	−0.075
	Sig. (two-tailed)	**0.007**	**0**	**0.002**	0.149	0.173	0.812	0.408
	*N*	122	123	119	124	123	124	124
HPL/creatinine	Correlation coefficient	0.091	0.021	0.061	0.064	0.098	−0.181	0.041
	Sig. (two-tailed)	0.299	0.813	0.493	0.465	0.266	**0.037**	0.637
	*N*	131	132	128	133	132	133	133
High DA ROC		0.244	0.213	0.136	0.13	−0.012	0.039	0.127
		**0.005**	**0.014**	0.125	0.135	0.887	0.659	0.145
		131	132	128	133	132	133	133
DA/HVA ROC		0.116	0.156	0.12	−0.1	0.189	0.044	0.041
		0.189	0.073	0.176	0.251	**0.03**	0.616	0.642
		131	132	128	133	132	133	133
High NA ROC		0.223	0.197	0.35	0.028	0.13	0.136	0.104
		**0.01**	**0.023**	**0**	0.746	0.137	0.118	0.232
		131	132	128	133	132	133	133
NA/NHMA ROC		0.162	0.106	0.26	0.079	0.125	0.088	0.034
		**0.067**	0.233	**0.003**	0.373	0.158	0.322	0.703
		128	129	125	130	129	130	130
High AD ROC		0.125	0.19	0.237	0.012	0.041	0.05	0.124
		0.156	**0.029**	**0.007**	0.894	0.644	0.57	0.156
		131	132	128	133	132	133	133
AD/MHMA ROC		0.075	0.199	0.21	−0.06	0.046	0.001	0.009
		0.403	**0.024**	**0.019**	0.501	0.608	0.99	0.922
		128	129	125	130	129	130	130

**Table 4:** **Total sample correlation matrix for biochemistry variables and ROCs in relationship to each other**.

ROC biochemistry variable correlates		Low VitD ROC AUC = 0.65	Low RC folate ROC AUC = 0.654	Low B6 ROC AUC = 0.638	High Se B12 ROC AUC = 0.565 (Sig 80%)	High Free Cu:Zn × 100 ROC AUC = 0.611	MTHFR homozygous	High HIST ROC AUC = 0.576
pl HCY ROC AUC = 0.557 (Sig 75%)	Correlation coefficient	0.13	0.179	0.063	−0.051	−0.045	−0.002	−0.036
	Sig. (two-tailed)	0.138	**0.039**	0.481	0.558	0.605	0.986	0.677
	*N*	132	133	129	134	133	134	134
Low VitD ROC AUC = 0.65	Correlation coefficient	1	0.239	0.055	−0.012	−0.038	−0.096	0.129
	Sig. (two-tailed)		**0.006**	0.54	0.894	0.669	0.275	0.141
	*N*	132	131	127	132	131	132	132
MTHFR_homozygous	Correlation coefficient	−0.096	−0.004	0.028	0.062	−0.107	1	−0.02
	Sig. (two-tailed)	0.275	0.967	0.753	0.473	0.22		0.823
	*N*	132	133	129	134	133	134	134
High HIST ROC	Correlation coefficient	0.129	0.029	−0.116	0.153	−0.054	−0.02	1
	Sig. (two-tailed)	0.141	0.738	0.191	**0.077**	0.534	0.823	
	*N*	132	133	129	134	133	134	134
Low RC folate ROC		0.239	1	0.262	−0.029	0.011	−0.004	0.029
		0.006		**0.003**	0.741	0.899	0.967	0.738
		131	133	128	133	132	133	133
High Se B12 ROC (Sig 80%)		−0.012	−0.029	−0.26	1	−0.061	0.062	0.153
		0.894	0.741	**0.003**		0.486	0.473	0.077
		132	133	129	134	133	134	134
Low B6 ROC		0.055	0.262	1	−0.26	0.171	0.028	−0.116
		0.54	**0.003**		**0.003**	**0.053**	0.753	0.191
		127	128	129	129	129	129	129
High Free Cu:Zn × 100 ROC		−0.038	0.011	0.171	−0.061	1	−0.107	−0.054
		0.669	0.899	**0.053**	0.486		0.22	0.534
		131	132	129	133	133	133	133

**Table 5:** **Structural equation model (SEM) for 134 variable of interest and biochemistry including FAD as a putative marker**.

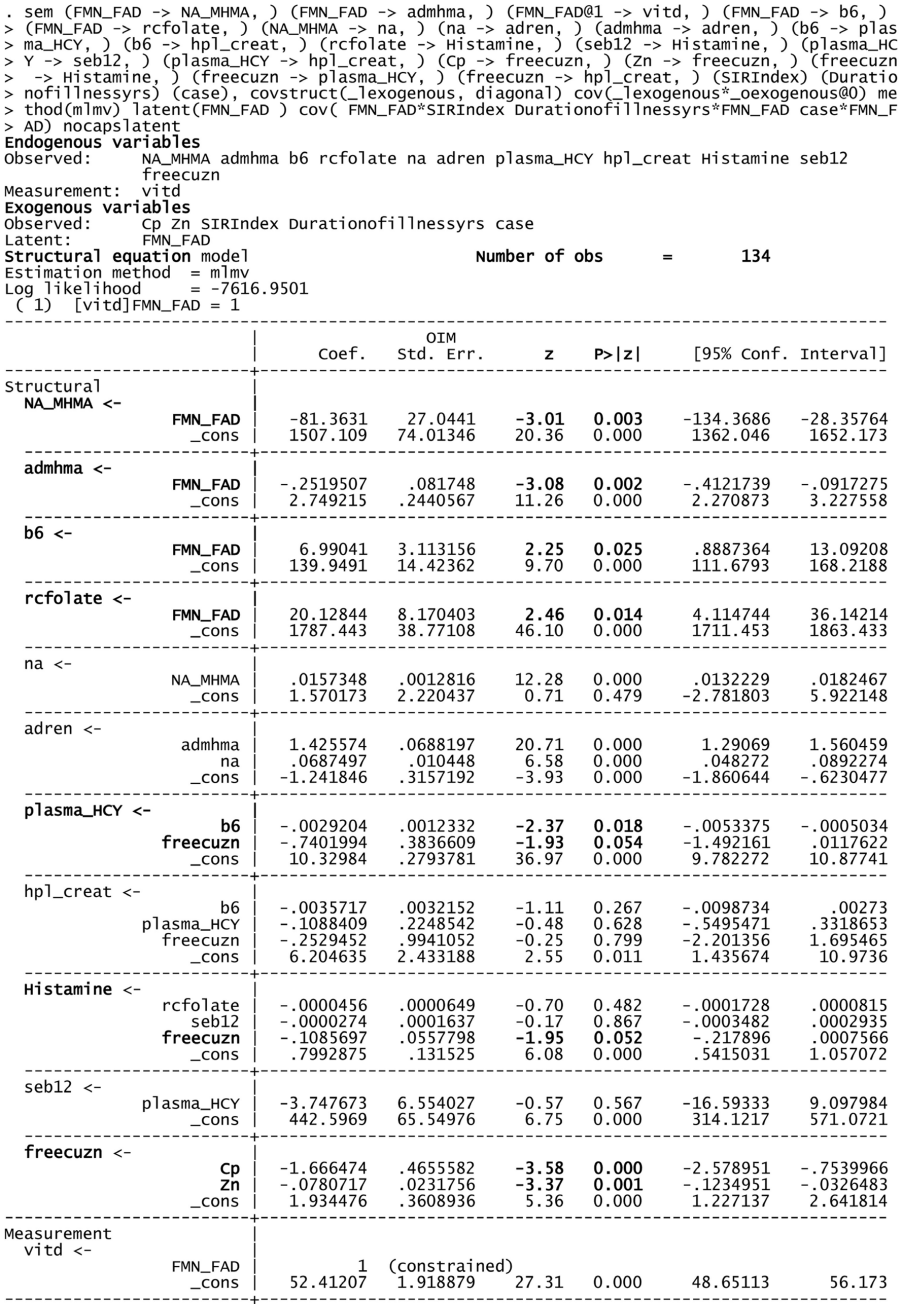
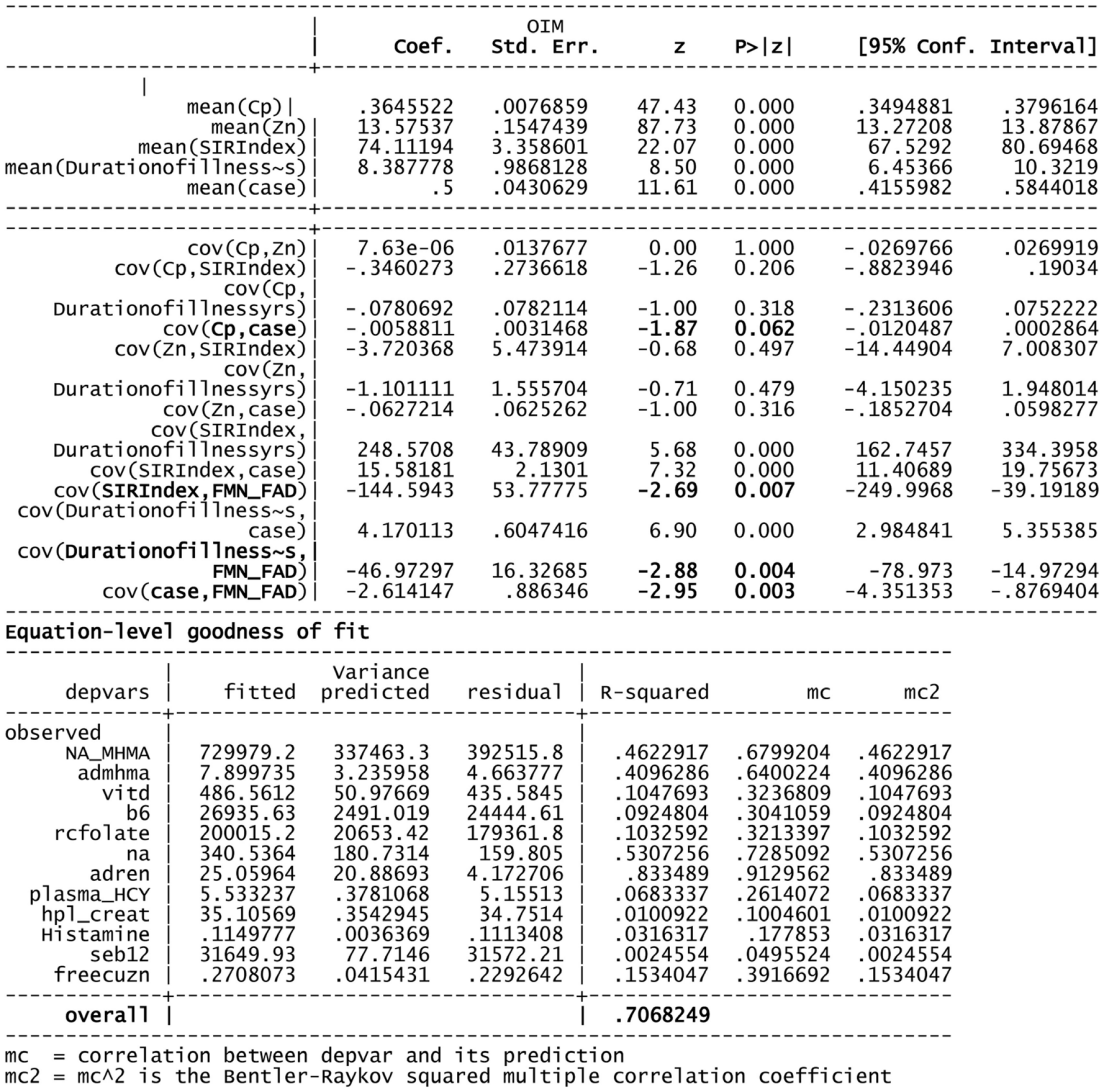

**Table 6:** **Key enzymes utilizing FMN or FAD in the biochemistry of schizophrenia or schizoaffective psychosis**.

FMN- or FAD-dependent enzymes	Role	Reference
Methyl tetrahydrofolate reductase (MTHFR)	Rate-limiting enzyme of the methylation cycle encoded by the MTHFR gene. Produces 5 methyl tetrahydrofolate (5MTHF) required to complete a turn of the folate cycle and allow methyl groups to enter the methionine cycle and homocysteine to be metabolized to Methionine.	(18)
Monoamine oxidase (MAO)	Utilized FAD as cofactor in first step metabolism of DA, NA, and AD. In this process, NA and AD are metabolized to MHMA	(19)
Diamine oxidase (DAO) (histaminease)	Metabolism of histamine contains FAD and Cu moieties. Found in the digestive tract, placenta, and secreted by eosinophils	(20)
Glutathione reductase (GSSH reductase) (6) Thought related to flavin reductase and ferrodoxin reductase enzymes (see below)	Restores glutathione (GSH) to its active, reduced form, afte the its metabolism to oxidized Glutathione (GSSH) by glutathione peroxidase. GSH serves as a major contributor to the redox balance in cells through its ability to scavenge and reduce reactive oxygen species (ROS)	(21)
Methyl synthase reductase (MSR) works in tandem with Methionine synthase (MS)	MSR reduces B12 back to its activated form (from II to I form) after use by methyl synthase (MS) enzyme which metabolizes homocysteine to methionine. In most tissues, this provides the sole pathway for homocysteine remethylation; therefore, MTHFR deficiency from MTHFR 677TT is associated with high plasma concentrations of homocysteine. Substrates for the MS enzyme are methionine synthase-cob(II)alamin, NADPH, H^+^, and *S*-adenosyl-l-methionine. And its products are methionine synthase-methylcobalamin, *S*-adenosylhomocysteine, and NADP	(22, 23)
Pyridoxal kinase (PdxK)	Pyridoxal phosphate is the active form of *vitamin B6. P*yridoxal kinase is required for the synthesis of pyridoxal-5-phosphate from vitamin B6, and catalyzes the conversion of pyridoxal to pyridoxal 5′-phosphate in the presence of ATP	(24)
Betaine homocysteine methyl transferase (BHMT)	Betaine = ttrimethyl glycine TMG). TMG degradation is a methylating pathway for methionine reconstitution utilizing homocysteine to produce dimethyl glycine (DMG) and methionine. DMG requires FAD to then de-methylates itself using mitochondrial dimethylglycine dehydrogenase (DMGDH). If FAD is deficient, DMG cannot de-methylate and the BHMT betaine degradation pathway is stalled.	(25, 26)
Flavin reductases (FR) (FMN–FAD reductase) Also called methemoglobin reductase. Has overlap function with 25 OH hydroxylase of vitamin D, Ferric reductase, and Biliverdin reductase b (see below). Also reduces the non-heme ferric center of ribonucleotide reductase and thereby influences DNA synthesis	Cytosolic enzymes that catalyze the reduction of FMN, FAD while allowing them to funnel electrons one at a time from NADH/NADPH to the ferrodoxin (Fe2+–S2+ protein) center of heme (with electron flow from NADPH to FAD to FMN to heme). In human erythrocytes. FR has high affinity for tetrapyrrole protoheme binding and reducing the iron center of methemoglobin. It also requires free flavins to catalyze the reduction of iron in order to release iron from storage or ferritin	(27, 28)
Biliverdin Reductase (Bilirubin-IXb reductase- isomer in erythrocytes)	Low B2, low B6, and low zinc combine to inhibit protoheme synthesis and B12 is also an inhibitor of Flavin reductase and FMN. Protoheme binds tightly to Bilirubin (Flavin) reductase to produce Biliverdin which in red calls is thought to be degraded by bilirubin IX reductase IXb to HPL. Where B2, B6, and zinc are low, protoheme synthesis is expected to be impaired and ALA is high	(29)
l-tryptophan pyrrolase [tryptophan 2,3-dioxygenase (TDO)]	A Heme flavoprotein cytosolic enzyme that catalyzes the first rate-limiting step of the kynurenic pathway- oxidative decarboxylation of L tryptophan with pyrrole ring cleavage, to produce NH_3_+CO_2_+ an unstable quinoxaline derivative that undergoes oxidative reduction with modification to resemble an HPL-like molecule. In a pseudomonas bacterium, the enzyme activity appeared to increase immediately after cessation of cell growth, responsible for anxiety-related behavior. TDO knockout mice had increased plasma levels of tryptophan, serotonin, and 5-HIAA in the midbrain and hippocampus	(30, 31)
Ferric reductase (FeR) Function also performed by heme c-type cytochromes in the membrane or periplasmic space (see below)	Iron can only be stored in ferritin in the ferric (Fe3+) state. FeR’s role is to deliver an adequate amount of safe ferric iron to the cells and may be an evolutionary adaptation to prevent toxic ferrous (Fe2+) leading to the Fenton reaction. FeR can transfer electrons from formate and FMN or FAD to form Fe+++ and Mn++++. Iron can only be stored in ferritin and transported by transferrin in the ferric (Fe3+) state. Ceruloplasmin also functions as a major ferroxidase in the blood, to hedge against any free iron in the circulation by converting iron to its ferric state that can be bound to transferrin. As such, it is majorly responsible for iron efflux from tissues, so when FAD is plentiful for FR function, ceruloplasmin is utilized in iron transport	(33–35)
25-OH-vitamin D3 hydroxylase	Mitochondrial flavoprotein monooxygenase contains cytochrome P 450 heme component and an iron sulfur ferredoxin protein. In a three component monooxygenase enzyme system, it regulates vitamin D3 activation using flavin-dependent ferrodoxin reductase and adrenodoxin reductase. Production of Vitamin D3 is feedback regulated by parathyroid hormone. 1-25 OH D3 necessary for full inflammatory response in macrophages. Is a mixed function oxidase, similar to adrenal cortex mono-oxygenases (ferredoxin type protein adrenodoxin and adrenoxin NADP+ reductase), concerned with steroidogenesis and with and P450-dependent monooxygenases	(36, 37)
P450 monooxygenases (P450 CYP4A4 responsible for 50% drug metabolism)	Flavin-dependent heme protein enzyme P450 monooxygenase enzymes require their heme iron to receive electrons from a flavin redox partner. Flavin electrons are transferred to the substrate, binding it. Once the substrate is bound, Fe within the heme (pyrrole) molecule is reduced.	(38)

**Table 7:** **MTHFR 677 split-sample correlates for psychosis caseness and functional measures, at 95% CI**.

Homozygous MTHFR polymorphism	Biochemical parameter	*N*	Rho	*P*	Wild-type enzyme (No MTHFR gene polymorphism)	Biochemical parameter	*N*	Rho	*P*
Psychosis caseness	HPL/creat	7	0.720	**0.067**	Psychosis caseness	NA	65	0.634	**0**
	Free	7	0.841	**0.018**		HIAA	65	0.475	**0**
	Cu/Zn					HPL/creat	65	0.33	**0.007**
	No HIAA					Vitamin B6	63	−0.335	**0.007**
						RC folate	64	−0.245	**0.051**
						Vitamin D	64	−0.245	**0.005**
Duration of Illness	High DA ROC	7	0.068	0.081	Duration of illness	NA	60	0.661	**0**
	HPL/creat	7	0.808	**0.028**		HIAA	65	0.403	**0.001**
	No HIAA					HPL/Creat	60	0.306	**0.017**
						Vitamin B6	58	−0.368	**0.004**
Symptom Intensity Rating (SIR)	DA	7	0.749	0.053	Symptom Intensity Rating (SIR)	DA	65	0.3	**0.015**
	HPL/creat	7	0.768	0.044		NA	65	0.498	**0**
	Free Cu/Zn	7	0.729	0.063		AD	65	0.508	**0**
	High Free Cu/Zn ROC	7	0.676	0.096		HIAA	65	0.403	**0.001**
	Low histamine <0.035 μg/l	7	0.676	0.096		HPL/Creat	65	0.341	**0.005**
	No HIAA					Vitamin D	64	−**0.288**	**0.021**
						Low RC folate ROC	64	0.217	0.086
Clinical Global Illness severity (CGI)	High DA ROC	7	0.794	**0.033**	Clinical Global Illness severity (CGI)	NA	58	0.596	**0**
	DA	7	0.896	**0.006**		HIAA	58	0.509	0
	Free Cu/Zn	7	0.717	**0.07**		HPL/creat	58	0.223	0.092
	No HIAA					RC folate	57	−0.344	**0**
						Vitamin B6	56	−0.359	**0.007**
						Vitamin D	57	−0.255	**0.055**
Global Assessment of Function (GAF)	Free Cu/Zn	7	−**0.741**	**0.057**	Global Assessment of Function (GAF)	NA	58	−0.522	**0**
	No HIAA					HIAA	58	−0.483	**0**
						HPL/creat	58	−0.297	**0.024**
						Vitamin B6	56	0.273	**0.042**
						Vitamin D	57	0.297	**0.025**
Social and Occupational Assessment Scale (SOFAS)	Nil				Social and Occupational Assessment Scale (SOFAS)	DA	58	−0.283	**0.031**
	NO HIAA					NA	58	−0.593	**0**
						RC folate	58	−0.287	**0.029**
						HPL/creat	58	−0.261	**0.048**
						Vitamin D6	56	0.364	**0.006**
						Vitamin D	57	0.255	**0.055**

### Further Results

When results from the full sample correlation analysis (Tables 2–4) were considered together with NA/MHMA and AD/MHMA ROCs (S8), (that reflected catecholamine metabolism by MAO) these ROCs were found significantly related to elevated NA (*n* = 133, rho = 0.673, *P* = 0.0000), elevated AD (*n* = 130, rho = 0.712, *P* = 0.0000), respectively (S7) (1). NA/MHMA and AD/MHMA ROCs were also significantly related to case-ness for psychosis (*n* = 133, rho = 0.505, *P* = 0.000) and (*n* = 130, rho = 0.483, *P* = 0.000), respectively. Moreover, if low SAMe levels and COMT inhibition due to low MTHFR activity with reduced 5MTHF methylation product and ensuing insufficient SAMe production for histamine-metabolism, were to be the major explanation for elevated NA and AD, then both of their should also hold a significant correlative relationship with high histamine (but they did not) (Figure 1; Table 3). For these reasons, there must be some other factor operating in functional psychosis to explain the low catecholamine turnover by the metabolizing enzyme MAO. Since the other factor likely to influence MAO turnover is an absolute or functional deficiency of its co-factor flavin adenine nucleotide (FAD), it was reasonable to assume that FAD insufficiency or its precursor Flavin adenine mononucleotide (FMN) may constitute an exogenous, unobserved variable influencing basement biochemistry of schizophrenia and schizoaffective disorder. Since FAD and its precursor FMN are derivatives of Riboflavin (Vitamin B2) riboflavin deficiency may also play an important role in the basement biochemistry of functional psychosis (see discussion section). Accordingly, SEM of the biochemistry data was undertaken to understand how FMN–FAD might relate to other dependent variables of significance.

Using FMN–FAD as the independent variable, the SEM model result is shown in Table 5. Overall goodness of fit (R2) for this model was 0.707, meaning that FMN–FAD explains 71% of the variance of the dependent biochemical variables. The SEM model also satisfyingly confirmed that significant negative covariance *z* scores exist between FMN–FAD and:

– Caseness for functional psychosis (*n* = 134, *z* = −2.95, *P* = 0.003),
– Symptom Intensity Rating (*n* = 134, *z* = −2.69, *P* = 0.007), and
– Duration of Illness (DOI) (*n* = 134, *z* = −2.88, *P* = 0.004).

The below SEM results imply that low levels of FMN–FAD are strongly related to high case ness, high symptom intensity (SIR) and longitudinal duration of illness (DOI) in schizophrenia and schizoaffective disorder, in the participant sample of this study. Though the valence of the *z* coefficient in the SEM model is determined to some extent by the model structure, given the goodness of fit of the model, it was interesting to observe the strength of significant *z* coefficients within the model. Their implications discussed in the discussion section, below.

### Collated Results of Correlative and SEM Analysis for Individual Variables within the Full Participant Sample

#### Total Sample Correlation and SEM Analysis Results Imply FMN–FAD Insufficiency Linked to Elevated Catecholamine Levels

– caseness and NA/MHMA (*n* = 130, rho = 0.514, *P* = 0.000).
– caseness and NA/MHMA ROC (*n* = 133, rho = 0.505, *P* = 0.000).
– caseness and AD/MHMA (*n* = 130, rho = 0.503, *P* = 0.000).
– caseness and AD/MHMA ROC (*n* = 130, rho = 0.483, *P* = 0.000).
– On SEM analysis, inverse findings were found between FAD and NA/MHMA (*n* = 134, *z* = −3.01, *P* = 0.003), FAD and AD/MHMA (*n* = 134, *z* = −3.08, *P* = 0.002), NA/MHMA and AD/MHMA represent the activity of the MAO enzyme in this study. This enzyme metabolizes NA and AD to MHMA. The close correlative relationship between caseness for functional psychosis and elevated NA/MHMA and AD/MHMA levels were confirmed on ROC biomarker analysis and again on SEM analysis, where robust inverse *z* coefficient s were found between FMN–FAD and NA/MHMA and FMN–FAD and AD/MHMA. Taken together, this means that: in a setting of low riboflavin availability MAO activity is low, leading to conserved, catecholamine elevation. Such MAO inhibition may relate more to -type B than type A form of the enzyme, since, in the periphery (from where urine samples for NA and AD are derived); these catecholamines are solely metabolized by the type B MAO enzyme. By contrast, DA may be metabolized by either type A or B form of MAO, possibly explaining why NA and AD elevations predominate over elevated DA findings in this study. Another explanation for the predominance of NA in this study, may also relate to animal study reports of intraluminal gastrointestinal bacteria, such as *Escherichia coli*, being a source of NA catecholamine (39). This imputed finding of FMN–FAD deficiency in relationship to MAO (40) and associated elevated catecholamines concur with multiple previous literature reports of riboflavin deficiency and MAO inhibition in behavior disorders, schizophrenia and schizoaffective disorder (12, 41–43).

#### Total Sample Correlation and SEM Analysis Results Imply FAD Insufficiency Linked to Low Vitamin D

– Low vitamin D levels yielded a sufficiently strong AUC to be included in the nutritional biochemistry bracket of biomarkers as a biomarker for functional psychosis (S6)
– Psychosis caseness correlated highly with vitamin D levels.
– Vitamin D levels correlated highly with all functional ratings for disability, caseness, and symptom intensity (SIR) (Table 1).
– Vitamin D levels inversely correlated with both NA and AD levels and their MAO metabolism markers NA/MHMA and AD/MHMA in a context where
– NA/MHMA and AD/MHMA both demonstrated significant inverse correlates with vitamin D (and also with folate and activated B6) (Table 2), in a setting where
– Vitamin D levels correlated positively with levels for vitamin B6 and folate.

In this study, vitamin D held significant correlates with respect to caseness, severity, and disability. Following on from vitamin D synthesis, FAD is required as a cofactor for vitamin D conversions to its active metabolic form of 25 (OH) vitamin D (44), which was the form of vitamin D assayed in this project. Furthermore, vitamin D levels and its ROCs indicating lower levels of vitamin D in cases than controls, correlated most closely with biomarker domains for impaired visual dysfunction and auditory dysfunction in psychosis (Table 4), both of which also held high correlates for disability and severity measures in the psychosis condition (Table 2). Vitamin D levels relate with greater inverse strength to elevated catecholamines (NA and AD) than to their metabolic ratios (NA/MHMA and AD/MHMA), which fits an alternative hypothesis that elevated catecholamines directly invoke cyclic adenosine monophosphate (cAMP) gene expression and cAMP induction of parathyroid hormone (45). The role of parathyroid hormone has been implicated in schizophrenia (46) and it releases calcium from cells that must then be replaced by the action of vitamin D. Such a situation may, therefore, result in overutilization of vitamin D with osteoporosis as also reported in the schizophrenic condition (47).

#### Total Sample Correlation and SEM Analysis Results Imply FAD Insufficiency and Low Folate

– The folate marker in this study yielded a sufficiently strong AUC to indicate that low levels of folate is a biomarker for functional psychosis (S6).
– Psychosis caseness correlated highly with the folate ROC variables (Table 3)
– Red cell folate level and its ROC correlated with all disability functions, caseness, and symptom intensity measures (Tables 2 and 3).
– Folate levels inversely correlated with both NA and AD levels and their MAO metabolism markers NA/MHMA and AD/MHMA in a context where
– NA/MHMA and AD/MHMA levels both demonstrated significant inverse correlates with folate levels (Table 2)
– Low folate ROCs correlated most closely plasma with low HCY and low vitamin D ROCs (Table 3) and folate and vitamin D levels were significantly correlated.
– On SEM analysis, FMN–FAD levels and red cell folate levels were significantly linked (*n* = 134, *z* = 2.46, *P* = 0.014) (Table 6).
– Folate levels correlated positively with vitamin D and vitamin B6 levels all of which formed deficit biomarkers in this study.
– Given the flow-on effect of low folate and 5MTHF on the methylation cycle, there was an expected strong correlative link between total low folate levels and HCY levels (Table 2) (48).

Apart from low folate absorption levels related to riboflavin-deficiency-induced gut inflammation (49), riboflavin is required for biosynthesis of folate coenzymes without which lower levels of useful folate are found in animal studies (50). In this study, major folate derivatives were assayed in the folate test, so inhibition of the MTHFR enzyme by insufficient FAD coenzyme was included in the inability of this enzyme to provide sufficient 5MTHF (51, 52). This would further explain the relationship found between FAD and red cell folate on SEM analysis.

#### Total Sample Correlation and SEM Analysis Results Imply FMN Insufficiency Linked with Low Activated B6

– Low B_6_ levels yielded a sufficiently strong AUC to be included as a biomarker for functional psychosis (S6) and psychosis caseness correlated highly with vitamin B_6_ ROC (Table 3).
– The low B6 ROC correlates highly with all disability functions and symptom intensity measures (SIR) (Tables 2 and 3).
– B_6_ ROC correlate marginally with Free Cu/Zn ROC (*n* = 129, rho = 0.171, *P* = 0.053)
– On SEM analysis, a positive coefficient was found between FAD and activated B6 (*n* = 134, *z* = 2.25, *P* = 0.025), with
– a significant negative relationship found between activated B6 and plasma HCY (*n* = 134, *z* = −2.37, *P* = 0.018) in a setting where B6 levels correlate inversely with serum B12 (*n* = 129, rho = −0.260, *P* = 0.009) (Table 2) and low vitamin B6 ROC correlates closely with high serum B12 ROC (Table 3).
– B6 levels and held significant inverse correlates with all catecholamine parameters and the low vitamin B6 ROC correlated significantly with NA and AD, but not with DA.

Pyridoxine 5′-phosphate oxidase is a FMN-dependent enzyme that converts vitamin B6 into its activated form -pyridoxine 5 phosphate (PLP), as was assayed in this study (53). In FMN insufficiency, activation of B6 is impaired (24); therefore, *S*-adenosylhomocysteine-hydrolase (SAHH) activation within the methylation cycle is retarded and *S*-adenosyl homocysteine (SAH) is conserved. Catecholamine metabolism by COMT activity is inhibited by high SAH (54) leading to conserved and elevated catecholamines. Within the catecholamine synthesis pathway, DOPA decarboxylase also requires activated B6 as a necessary cofactor for DA synthesis. If B6 activation is retarded by insufficient FAD, DA synthesis is also retarded, which explains the absence of correlates between high DA ROC and Low B6 ROC in a setting where there is overall COMT catecholamine conservation. Vitamin B6 is a necessary cofactor for several enzymes within the transulfuration pathway to glutathione synthesis. Its inactivity through insufficient FMN will also retard HCY metabolism to cysteine and so elevate HCY levels as well as contributing to reduced glutathione (GSH) production and oxidative stress. Given the relationship of B6 to this pathway and its participation in the heme synthesis pathway (55, 56), it was interesting that there was a notable lack of significant correlates found between vitamin B6 and the supposed oxidative stress and heme-degredation marker, HPL. This can perhaps be explained by the relationship found between the vitamin B6 and Free Cu/Zn ROCs, which implies that when unbound copper is elevated, vitamin B6 is also elevated. A possible explanation for this finding again directs attention to the transulfuration pathway where Cu inhibits the rate-limiting enzyme cystathione beta synthase (CBS) (57), leading to underutilization of vitamin B6, which is a necessary cofactor for at least four enzymes within this pathway (10).

#### Total Sample Correlation and SEM Analysis Results for Elevated Histamine

– High histamine ROC (*n* = 134, rho = 0.1641, *P* = 0.058) correlated with psychosis caseness in this study and
– NA+ histamine together form a high quality ROC (*n* = 134, rho = 0.839, *P* = 0.0001).
– However, no correlation strength was found between NA/MHMA ROC or AD/MHMA ROCs and high histamine ROC (Table 3), but
– a marginally significant negative relationship was found between histamine and free Cu/Zn (*n* = 134, rho = −1.95, *P* = 0.052) that was supported on SEM analysis by a negative coefficient between these two variables (*n* = 134, *z* = −1.95, *P* = 0.052).

Histamine is a neurotransmitter that creates wake-fullness and plays a role in local immune response. Histamine emerged as a low-grade biomarker in this study and together with NA, formed a very strong compound biomarker for functional psychosis with symptom correlates linked to anxiety, fear, and over-arousal in our patient sample (17). Inflammatory bowel disease releases histamine from mast cells in the gut lining, a hypothesis that is supported within the immune activation theory of schizophrenia (58, 59). SEM analysis demonstrated a negative relationship between histamine and free Cu/Zn, which will be discussed later in terms of low histamine findings. Though FAD did not directly relate to histamine on SEM analysis, insufficient FAD for good MTHFR activity may provide one explanation since undermethylating environments with insufficient SAMe available for the histamine-metabolizing enzyme *N* methyl histamine means that there is a tendency for histamine to be conserved and elevated (60).

#### Total Sample Correlation and SEM Analysis Results for HPL

– elevated HPL ROC and NA/MHMA (*n* = 133, rho = 0.205, *P* = 0.018).
– elevated HPL ROC and AD/MHMA (*n* = 130, rho = 0.224, *P* = 0.010).
– HPL levels and vitamin B12 levels both correlated with the symptom intensity index (SIR) (Tables 2 and 3) (17).
– HPL/creatinine has an inverse relationship with the MTHFR homozygous 677TT polymorphism (*n* = 133, rho = −0.181, *P* = 0.037).
– SEM analysis found no significant relationship between HPL and FMN–FAD.

The oxidative stress marker in this study, heme-hydroxyl pyrroline-2-one (HPL), has been reported in the urine of persons suffering from schizophrenic psychosis (4). In this study, significant correlates were found between NA/MHMA and AD/MHMA and elevated HPL/creatinine levels in a setting where high HPL levels formed a biomarker for functional psychosis and held high correlation with psychosis symptom intensity index (SIR) (17). The closest correlative relationship for HPL was an inverse one with the MTHFR homozygous polymorphism, the significance of which is discussed in the next section.

**Figure 3:**
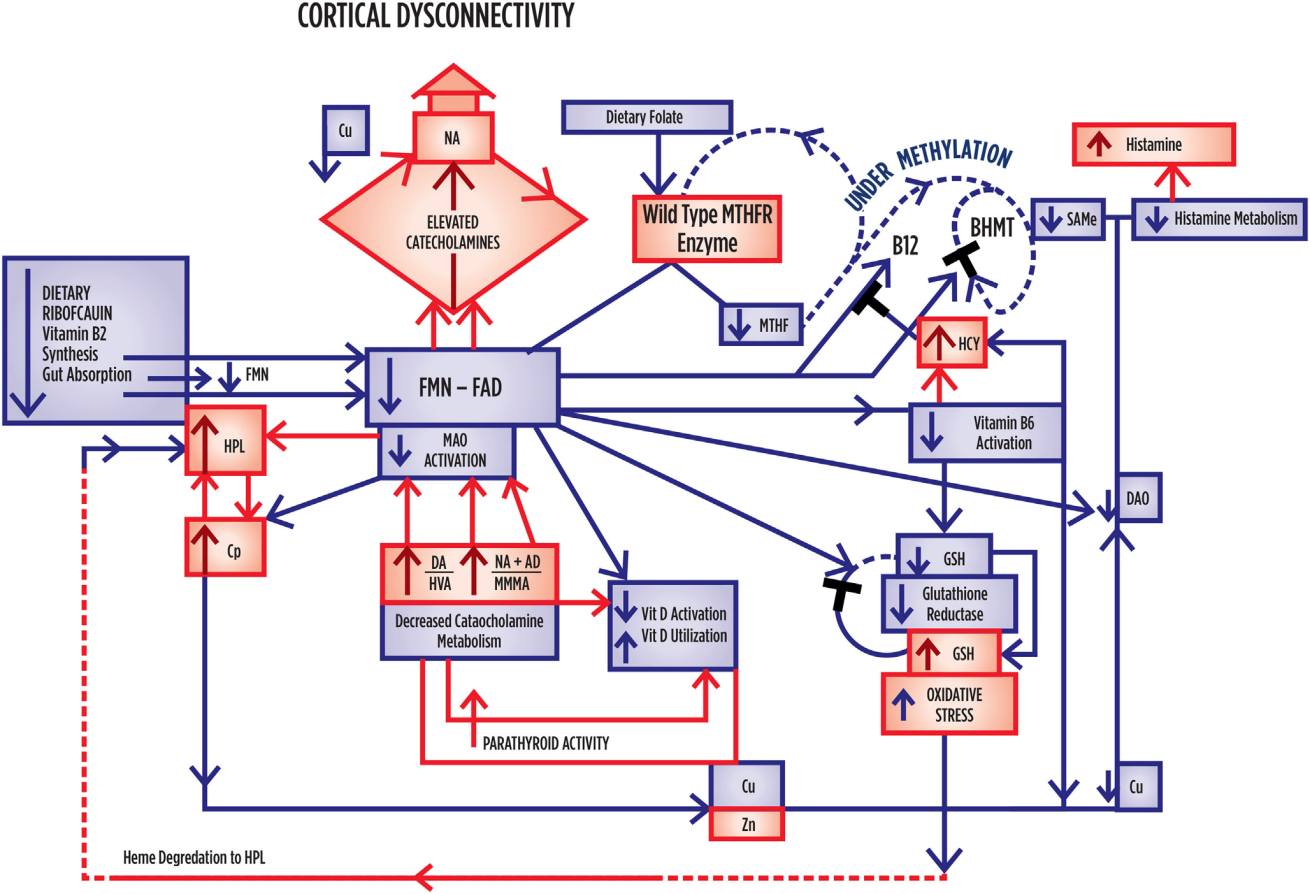
**Summary outcome when there is no MTHFR Polymorphism governing activity of the MTHFR enzyme, and where folate, vitamins, and flavins are low**. Where correlation significance index [SI] = [(1 – *P*-value) × 1000], caseness for schizophrenia or schizoaffective disorder relates to NA [SI = 100] and HPL/Creatinine [SI = 93], in a setting where catecholamines held significant correlates with the wild-type enzyme and where NA relates to SIR [SI = 100], GSI [SI = 100], DOI [SI = 100], and NA relates to NA/MHMA [SI = 100]. With respect to vitamin levels, Low Vitamin B6 relates to SOFAS [SI = 94], CGI [SI = 93], and DOI [SI = 86]. Low vitamin D relates to SIR [SI = 79], CGI [SI = 59], SOFAS [SI = 45], and low folate levels relate to Psychosis Caseness [SI = 49]. All catecholamines formed highly significant positive correlates with HPL. All catecholamines held significant inverse correlates with folate, vitamin B6 and vitamin D. Free copper to zinc ratio formed no correlates of significance within this data-set. Within the data set, homocysteine held significant inverse correlates with folate and vitamin B6. **UNDER-METHYLATION DIMENSION CHARACTERISTICS. HIGH oxidative stress levels. HIGH catecholamine levels, where elevated DA and NA predominate. LOW vitamin B6, D and folate levels. LOW unbound Cu levels with tendency to high DA. Tendency to higher histamine and higher 5 HIAA excretion**.

Glutathione reductase is a FAD-dependent enzyme, which is sensitive to riboflavin deficiency (61). This enzyme lies at the bottom of the transulfuration pathway and requires FAD cofactor for the utilized, oxidized form of glutathione (GSSH) to re-constitute itself back to its reduced active (GSH) form in order to be useful in antioxidant defense (Figure 1) (62). In the absence of synthesis of GSH in the transulfuration pathway, the effects of heme-oxygenase in the first step toward heme synthesis would be expected to lead to increased downstream porphyrin synthesis and heme formation. However, other enzymes in this heme- synthesizing pathway, such as the critical enzyme d-aminoaluvenicacid (ALA) synthase (63), are also retarded by insufficient activated B6. In such a setting, low heme production and lower antioxidant bilirubin levels would be expected to lead to metabolic crisis and neuronal death (64), Amid such an oxidative-heme crisis, heme’s porphyrobilinogen side chain is perported to be decarboxylated and deaminated to form a labile hydroxylactam hemopyrrole fragment that corresponds to the hydroxyhaemopyrroline-2-one (HPL) molecule that is a biomarker in this study (4). In such a low heme synthesis context, it is possible that heme components within multiple heme-flavo-enzymes, such as tryptophan pyrrolase [tryptophan 2, 3-dioxygenase (TDO)] and P450 monooxygenase enzymes, may also be degraded to form HPL (Table 6). Since linear tetrapyrroles, such as bilirubin, play a significant role as antioxidant and anti-inflammatory agents, the reduction of protoheme synthesis by B6 unavailabilty and its further degradation to bilirubin allows further vulnerability to inflammation and oxidative stress. Oxidative damage to cells is in turn accompanied by kinin release precipitating an inflammatory acute phase reaction with elevated acute phase reactants, such as ceruloplasmin (Cp) and increased bound copper. In such a case, free (unbound) copper may be insufficiently available for DAO and MAO activity (65, 66) and for DA carboxylase conversion of DA to NA (67). This will respectively predispose to elevated histamine, elevated catecholamine and conserved DA, to produce the kind of high DA, elevated neurotransmitter profile that accompanies hyperactive, manic symptoms (17, 68) (Figure 3).

## Summary and Discussion of Findings for Correlation and SEM Analysis of the Full Sample

In this study, intra-class correlative variables for catecholamines were found to conform to known relationships for their established pathways for synthesis and metabolism. Furthermore, catecholamine/metabolite ratios indicating abnormal monoamine oxidase activity, could not be explained without imputing riboflavin-related compounds, such as FMN and FAD as intermediary variables. SEM analysis then confirmed that riboflavin-derived FMN–FAD played a 71% role in connecting variables within the biochemical pathways of interest. In this study, low folate and low activated vitamin B6 levels were also found related to both caseness and disability measures for functional psychosis (Tables 2 and 3), and SEM analysis confirmed their significance, and relatedness to FMN-FAD, by finding significant positive *z* coefficients between FMN–FAD and red cell folate and FMN–FAD and activated B6.

Riboflavin (vitamin B2) is a water-soluble vitamin derived from whole grains, wheat germ, wild rice, mushrooms, soybeans, brewer’s yeast, milk, yogurt, eggs, broccoli, Brussels sprouts, and spinach. Riboflavin serves as the precursor of FMN, which is the further precursor of FAD (69). In a riboflavin kinase reaction regulated by thyroid hormone (Figure 1) (70), riboflavin is transformed to FMN by the phosphorylation and FAD is then formed by the action of a second enzyme, FAD synthase. In riboflavin deficiency, plasma concentrations of FMN and FAD are, therefore, lowered (71, 72).

Stress is both a risk factor and inherent component of schizophrenia (73, 74) and it is well-known that the chaotic lifestyle accompanying psychosis is associated with stress and poor dietary vitamin intake. Low riboflavin related to poor diet in schizophrenia (75) may enhance susceptibility to low grade bowel inflammation and poor vitamin absorption (76) In addition, chronic stress with high cortisol levels has been found to reduce intestinal absorption of riboflavin (77). Moreover, riboflavin deficiency is reported associated with impaired maintenance of mucous membranes and low grade bowel inflammation (78, 79) which could well contribute to the malabsorption of nutrients such as B6, zinc, and folate (80). In this regard, individuals with schizophrenia have reported gastrointestinal problems and inflammation markers (81, 82), in a setting where diet changes and dysregulation of gut flora has also been associated with immune and inflammatory conditions (83). Moreover, in animal studies, riboflavin supplementation has been demonstrated to improve zinc absorption (84) and a marker of abnormal gut mucosal integrity has been reported linked to elevated HPL levels (85). This result could indeed be expected if subclinical riboflavin-deficiency from poor diet or disturbed gut microbiome, renders the gut mucosal wall unable to maintain its surface mucosal integrity, resulting in the kind of gastrointestinal inflammation and immune activation that has been reported in schizophrenia. For these reasons, riboflavin may occupy a critical position in the link between high schizophrenia rates, immune activation with inflammation in famine conditions, where grains and meat are difficult to procure (86).

**Figure 4:**
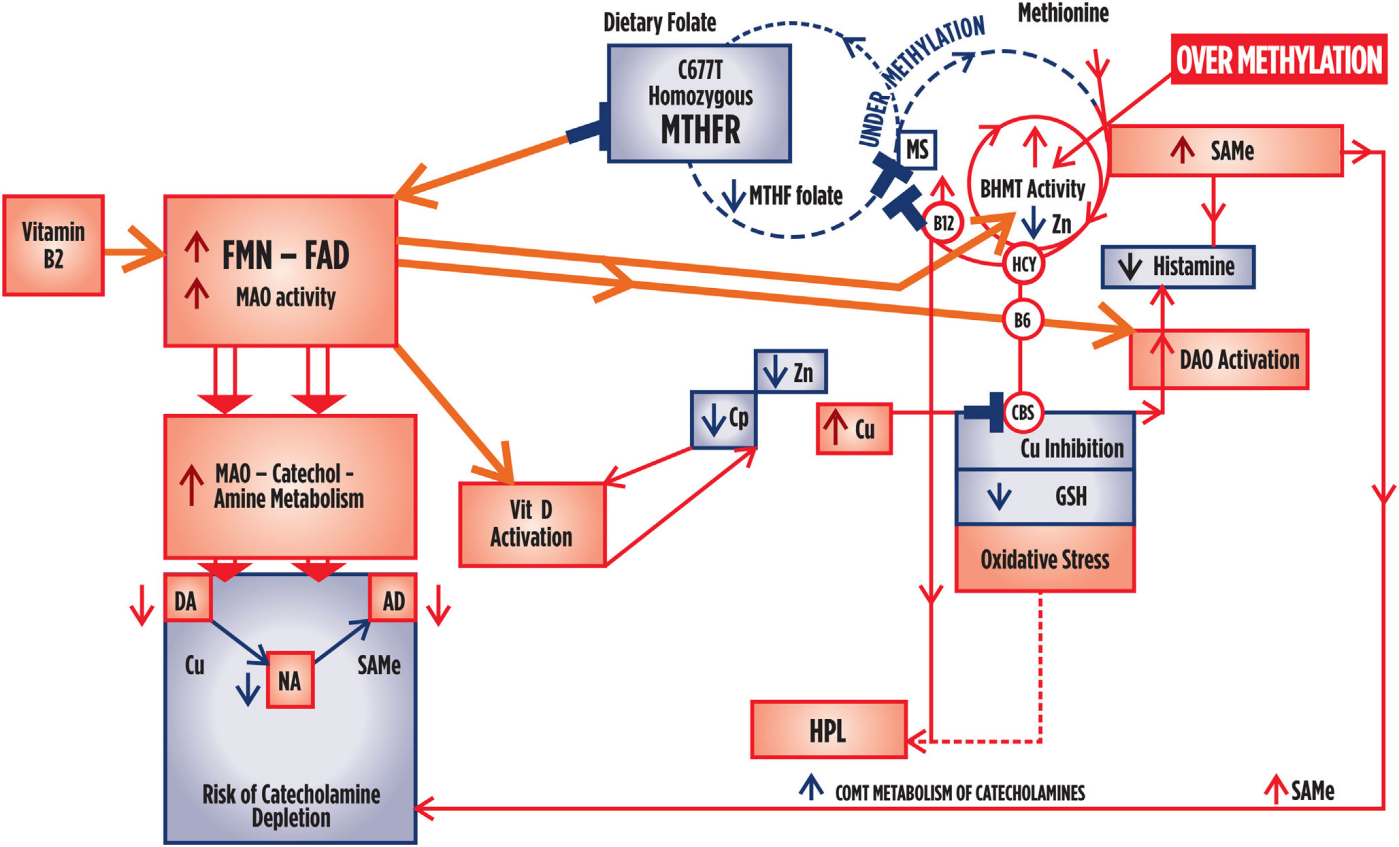
**Summary outcome when there is a homozygous 677TT polymorphism governing low activity of the MTHFR enzyme, but where folate and flavin availability in the presence of normal hormonal conditions and sufficient sources of flavins and folate from dietary and gastrointestinal sources**. Where significance index [SI] = [(1 − *P*-value) × 1000], caseness for psychosis is only marginally related to HPL/Creatinine [SI = 33]. Caseness was also marginally related to Free Cu/Zn ratio [SI = 33], where Free Cu/Zn also relates to GAF [SI = 43] and CGI [SI = 30]. Catecholamine held no significant correlates within this data set. Levels of folate are low in relationship to high homocysteine [SI = 84], and B12 [SI = 45] and high homocysteine levels correlate with low B6 levels [SI = 14]. There is no inference in the correlates to suggest that vitamin D levels are low. Flavin sufficiency is implied. Less severe oxidative stress severity and disability compared to the wild-type enzyme. High Cu levels promote DA metabolism to NA, with tendency to lower DA than NA levels. Vitamin levels are normal or increased. Tendency to lower histamine and no 5HIAA excretion. **RISK OF OVER-METHYLATION EXISTS**.

Flavin adenine nucleotide is a necessary cofactor for activity of the MTHFR enzyme that manufactures 5-MTHF methyl groups for running the methylation cycle. When the MTHFR 667 C → T gene encoding this enzyme is in a homozygous (MTHFR 677TT) state (where thymidine fully replaces its cytosine residues at position 677 of the gene), the encoded enzyme encoded has impaired ability to manufacture sufficient 5 MTHF to supply the methylation cycle. 5MTHF participates in MS conversion of HCY back to methionine and subsequent production of the methyl donor SAMe. Within the MS reaction, FMN is also required for the conversion of cobalamin cofactor to its active form. Though most tissues prefer to utilize the MS HCY to methionine conversion pathway, an alternative HCY metabolism rescue pathway exists for use when MS activity is blocked by insufficient 5MTHF and FMN cofactors. In this alternative pathway, betaine degradation is initiated *via* betaine homocysteine methyltransferase (BHMT) (Figure 1); however, this enzyme may be retarded in Flavin insufficient states due to the fact that the second step in its pathway requires FAD for dimethyl glycine (DMG) HCY de-methylation and without sufficient FAD, DMG accumulates and the BHMT betaine degradation pathway is stalled (26, 87). Both vitamin D and vitamin B6 also require FMN or FAD for synthesis into their activated forms, as assayed in this study (24, 88) and the active (reduced) form of glutathione (GSH) also requires FAD as a cofactor (21). FMN–FAD-dependent enzymes are also required for MAO activity in metabolizing the first step of catecholamine metabolism and diamine oxidase (DAO) requires FMN for metabolism of histamine (19, 20). Other enzymes requiring flavin as cofactors or as internal moieties to assist their activity are ubiquitous in body and brain biochemistry. Many have mixed functions, as outlined in Table 6. Understanding the meaning of the results of this study should, therefore, be undertaken in conjunction with Table 6, and Figures 1, 3 and 4 which further display the potential role of Riboflavin, FMN, and FAD in the biochemistry of functional psychosis.

### Investigation of MTHFR 677TT Gene Status in Relationship to Key Variables Using a Split Data Set

The MTHFR gene was selected as a marker for examination in this study because this gene codes for the MTHFR enzyme which is the rate-limiting factor in the methylation cycle (18). In the normal form of this gene, cytosine is at position 677, leading to an alanine at amino acid 222. However when there is thymidine at position 677, there is a valine substitution at amino acid 222 and this homozygous form of the gene (677TT) encodes a thermolabile enzyme with reduced activity compared to individuals with the CC or CT (heterozygous) forms of the gene (89). Though there is ethnic variability related to this polymorphism, 10% of the North American population are T-homozygous for this polymorphism.

The MTHFR gene polymorphism has a reported relationship to schizophrenia (90) and the MTHFR enzyme coded by the 677TT gene loses its FAD cofactor three times faster than the wild-type protein (91). It was, therefore, decided to split the data set from this study into three data-sets based upon the three possible gene coded states of the MTHFR enzyme (wild type, heterozygous and homozygous types) and to examine key correlates within those three data sets. Though there were only seven participants in the study with the homozygous form of the 667TT gene, comparison of the data sets still yielded variables of significance and correlative results analysis are presented in Tables 7 and 8 with results summarized in Figures 3 and 4.

**Table 8:** **MTHFR 667 split-sample correlates for psychosis biochemistry at 95% CI**.

	Biochemical correlate	*N*	Rho	*P*
**Homozygous MTHFR polymorphism**				
DA level	NA	7	0.679	**0.094**
High DA ROC	NA	7	0.791	**0.034**
DA/HVA level	Nil sig			
NA Level	DA/HVA	7	0.786	**0.036**
High NA ROC	NA	7	0.791	**0.034**
NA/MHMA	DA/HVA	7	0.964	**0**
AD Level	nil sig			
High AD ROC	nil sig			
HPL/creat	Caseness	7	0.722	**0.067**
	SIR	7	0.768	**0.044**
	DOI	7	0.808	**0.028**
RC folate	Homocysteine	7	−0.739	**0.058**
Low red cell folate ROC	Homocysteine	7	0.874	**0.01**
Homocysteine	RC folate	7	−0.739	**0.016**
	Vit B6	7	−0.847	**0.016**
Vitamin B6	Homocysteine	7	−0.847	**0.016**
Low B6 ROC	Homocysteine	7	0.798	**0.032**
Serum B12	HPL/creat	7	0.679	0.094
Vit D level	Se Cp	7	0.771	**0.042**
Low Vit D	RC zinc	7	−0.964	**0**
ROC	Nil			
Free Cu/Zn	Caseness	7	0.722	**0.067**
	GAF	7	−0.741	**0.057**
Cp	Zinc	7	−0.716	**0.07**
	Vitamin D	7	0.771	**0.042**
RC zinc	Vitamin D	7	−0.964	**0**
Histamine ROC	Vitamin B6	7	0.866	**0.012**
**Wild-type enzyme (No MTHFR gene polymorphism)**				
DA level	DA/HVA	65	0.294	**0.017**
	NA	65	0.483	0
	HPL/creat	65	0.307	**0.013**
	RC folate	64	−0.234	**0.063**
	Vitamin B6	63	−0.257	**0.042**
	Vitamin D	64	−0.307	**0.002**
High DA ROC	DA/HVA	65	0.372	**0.001**
	NA	65	0.399	**0.006**
	HPL/creat	65	0.339	**0.004**
	RC folate	64	−0.352	**0.034**
	Vitamin D	64	−0.265	**0.031**
DA/HVA level	RC folate	64	−0.27	0.08
	Cp	65	0.219	
NA Level	Caseness	65	0.634	**0**
	HPL/creat	65	−0.238	**0.062**
	RC zinc	65	−0.247	**0.047**
	Vitamin D	64	−0.477	**0**
High NA ROC	HPL/creat	65	0.226	**0.032**
	Vitamin B6	63	−0.383	**0.002**
	RC zinc	65	−0.238	**0.056**
	Vitamin D	64	−0.326	**0.009**
NA/MHMA	NA	63	0.82	**0**
	Vitamin B6	61	−0.227	**0.078**
	RC zinc	63	−0.23	**0.065**
	Vitamin D	64	−0.36	**0.004**
AD level	NA	65	0.743	**0**
	HPL/creat	65	−0.241	**0.053**
	Vitamin D	64	−0.361	**0.003**
High AD ROC	NA	65	0.69	**0**
	Vitamin D	64	−0.345	**0.005**
HPL/creat	Caseness	65	0.33	**0.007**
	NA	65	0.233	**0.062**
	Cp	65	0.262	**0.035**
	RC zinc	65	−0.261	**0.035**
RC folate	Caseness	64	−0.245	**0.051**
	DA/HVA	64	−0.27	**0.031**
	Homocystein	63	−0.26	**0.036**
	Se B12	64	0.226	0.072
	Vitamin B6	62	0.287	**0.024**
	Vitamin D	63	0.295	**0.019**
Low red cell folate ROC	Homocystein	63	0.283	**0.025**
	Vitamin D	63	−0.016	**0.018**
Homocysteine	RC folate	63	−0.264	**0.036**
Vitamin B6	NA	63	−0.384	**0.002**
	RC folate	62	0.287	**0.024**
	RC zinc	62	0.293	**0.02**
Low B6 ROC	NA	63	0.45	**0**
	Se B12	63	−0.233	**0.021**
	RC folate	62	−0.233	0.068
	RC zinc	63	−0.302	**0.016**
	Vitamin D	62	−0.292	**0.024**
Serum B12	RC folate	64	0.226	0.072
	Vitamin B6	63	0.235	0.063
High Se B12 ROC	RC zinc	65	0.245	**0.05**
Vit D level	NA	64	−0.447	**0**
	RC folate	63	0.295	**0.019**
Low Vit D ROC	NA	64	0.393	**0.001**
	Se B12	64	−0.336	0.06
	Vitamin B6	63	−0.314	**0.013**
	RC zinc	64	−0.284	**0.023**
Free Cu/Zn	Cp	64	−0.218	0.083
	RC zinc	64	−0.325	**0.009**
Cp	HPL/creat	65	0.262	**0.035**
	DA/HPA	65	0.219	0.08
RC zinc	HPL/creat	65	−0.261	**0.035**
	B6	63	0.293	**0.02**
	Free Cu/Zn	64	−0.325	**0.009**
Histamine ROC	Free Cu/Zn	64	−0.212	0.093

### Split Data-Set Results for Wild-Type MTHFR Enzyme

For the sake of emphasis, significant correlates for between 63 and 65 variables in this part of the split data set (Table 8) were indexed for their level of significance using the formula Significance Index [SI] = [(1 − *P*-value) × 1000] with the following significance indices obtained:

Caseness for schizophrenia or schizoaffective disorder relates to NA [SI = 100] and HPL/Creatinine [SI = 93], in a setting where catecholamines held significant correlates with the wild-type enzyme and where NA relates to SIR [SI = 100], GSI [SI = 93] and DOI [SI = 93] and NA relates to NA/MHMA [SI = 93]. With respect to vitamin levels, low vitamin B6 relates to SOFAS [SI = 94], CGI [SI = 55], DOI [SI = 86]. Low vitamin D relates to SIR [SI = 79], CGI [SI = 55], SOFAS [SI = 45], and Low folate levels relate to psychosis caseness [SI = 49]. All catecholamines formed highly significant positive correlates with the oxidative stress marker, HPL. All catecholamines held significant inverse correlates with folate, vitamin B6 and vitamin D. Free Copper to zinc ratio formed no correlates of significance within this data-set. Within the data set, homocysteine held significant correlates with folate and vitamin B6.

Flavin adenine nucleotide is a necessary cofactor for vitamin D and vitamin B6 activation and the wild-type MTHFR enzyme is also sensitive to riboflavin status (24, 88, 92). In an unstable folate or FAD deprived setting, the amount of MTHFR’s product 5-methylTHF is reduced relative to other folate forms. In turn, the downstream enzyme methionine sulfoxide reductase (MSR) (Figure 1) is deprived of its 5MTHF substrate, bringing about stasis of its conjointly acting enzyme MS. MS converts HCY back to methionine to replenish the methylation cycle. This set-back in HCY metabolism is also retarded in a low Flavin environment by the inability of vitamin B12 to reactivate itself after use and continue to cofactor MS (93) (Figure 3). In addition, there is a supplementary enzyme BHMT which in normal circumstances can rescue any MS retarded HCY metabolism blockage by its product, DMG utilizing FAD for re-methylation. In a low FAD environment, this enzyme finds its DMG product increased and its activity inhibited (94). Such stasis in both MS and BHMT pathways within the methylation cycle results in reduced methionine reconstitution and reduced SAMe output (Figure 3). Then, the COMT enzyme which requires SAMe as a cofactor will be less able to metabolize catecholamines and elevated catecholamines will ensue. The net effect is that folate and methionine cycle stasis occurs creating an under-methylating state with reduced SAMe formation.

The direction of significant correlates obtained from the split data set for the wild-type MTHFR enzyme (Tables 8 and 9) are consistent with an under-methylating biochemistry profile within which FMN–FAD insufficiency explains the combined inter-relationships of elevated catecholamine levels, elevated oxidative stress (HPL) and low levels of folate, vitamin B6 and vitamin D. A significant correlate was also found for high Se B12 ROC in relationship to zinc, though due to the fact that MSA for which B12 cofactors is a zinc utilizing enzyme (95). It was also considered significant that despite free copper to zinc ratio forming an elevated biomarker in the overall sample set, no significant variables were found related to it in the split data set for the wild-type enzyme. This implies that elevated free copper is not a significant issue requiring dynamic consideration when the wild-type enzyme is present in schizophrenia and schizoaffective psychosis.

**Table 9:** **Summary comparison of two main MTHFR variant – biochemical types, based upon combined findings from correlation, SEM, and significance indices [SIs] for caseness (Tables 7 and 8; Figures 3 and 4)**.

Characteristic	MTHFR homozygous	MTHFR-no polymorphism
Flavins FMN-FAD	Sufficient or +	Low
Vitamin levels	Sufficient or +	Low
Methylation profile pattern	Sufficient or +	Low
Severity CGI	+	+++
Disability (GAF)	+	+++
Catecholamines	AD marginal	+++ (DA and NA predominate)
		With low folate ++
		With low vitamin B6 ++
		With low vitamin D ++
Noradrenaline	−	++++
5HIAA excretion	−	++
HPL oxidative stress marker	+	++++
High % free Cu/Zn ratio	+	−
	GAF +	With high DA +
	CGI +	
Low RC folate	+	++
	With high homocysteine ++	With psychosis caseness ++
	With high vitamin B12 +	
High homocysteine	+++	++
	With low vitamin B6 +	With low activated vitamin B6 +
		With low folate +
Low vitamin B6	−	++
		With low SOFAS ++
		With high CGI ++
		With DOI ++
Low vitamin D levels	−	++
		With SIR +++
		With CGI ++
		With low SOFAS ++
Serum histamine	−	++

Vitamin B6 is dependent upon FAD for its activation to pyridoxine 5 phosphate (PLP) and adequate levels of activated vitamin B6 are required for both serotonin synthesis by l-tryptophan hydrolase and metabolism of serotonin’s precursor substrate l-tryptophan in the first part of the kynurenic pathway. Activated B6 and FAD are also required further down this pathway for breakdown of kynurenic acid (96–98). Therefore, in a no polymorphism MTHFR setting with low FAD and consequent low activated vitamin B6, metabolism in the kyneurenic pathway is inhibited and l-tryptophan might be expected to be preferentially diverted into the serotonin synthesis pathway. This may explain the significant levels of serotonin metabolite 5HIAA found in this study when there is no MTHFR polymorphism coding for activity of the MTHFR enzyme.

### Aligned Full Sample, SEM, and Split Sample Results Related to the Homozygous MTHFR 677TT Polymorphism

In this study, a full sample data-set was used to obtain a background understanding of dynamics at work between biochemical and functional variables. SEM analysis then confirmed that riboflavin-derived FMN–FAD played a 71% role in connecting variables within the biochemical pathways of interest. Given this understanding, the data set was split to examine the influence of the wild-type 677C → T, homozygous 677TT, and heterozygous 677CT genes coding for the MTHFR enzyme, with respect to other biochemical variables of interest within the data-set. Dynamic understandings derived from these three different levels of data analysis were then collated and aligned in order obtain a broad perspective of the dynamics at work within the biochemistry of schizophrenia and schizoaffective psychosis.

In the full participant sample (*n* = 134), the homozygous 677TT MTHFR polymorphism had no significant ROC strength for differentiating cases from non-cases (*n* = 134, AUC = 0.537, *P* = 0.3577) (S7) and a non-significant negative correlate was found between caseness and this polymorphism (*n* = 134, rho = −0.335, *P* = 0.700).

– No correlative relationship was found between either elevated NA/MHMA or AD/MHMA ROCs and the homozygous 677TT MTHFR polymorphism.
– No significant correlation was found between the MTHFR homozygous polymorphism and any deficit of the vitamin markers for B6, vitamin D, or B12
– As expected (due to reduced MTHFR polymorphism products and HCY metabolism impairment), there was a positive correlation between the MTHFR homozygous polymorphism and HCY levels (*n* = 134, rho = 0.224, *P* = 0.009).
– There was an inverse correlate existing between the homozygous form of MTHFR and the high HPL/Creatinine ROC oxidative stress biomarker (*n* = 133, rho = −0.181, *P* = 0.037). This means that very elevated HPL levels indicative of oxidative stress are not associated with the homozygous MTHFR polymorphism.
– In keeping with other studies, correlations were found between the homozygous 677TT polymorphism and HCY levels (99, 100).

For emphasis, *P*-values within the split sample for MTHFR 677TT were again indexed for significance, using the formula Significance Index [SI] = [(1 − *P*-value) × 1000] and the following indices were obtained:

– Caseness for psychosis is only marginally related to HPL/creatinine [SI = 33].
– Caseness was also marginally related to Free Cu/Zn ratio [SI = 33], where Free Cu/Zn also relates to GAF [SI = 43] and CGI [SI = 30].
– Apart from expected inter-relationships between catecholamine entities themselves, as a whole they held no significant correlates within this data set.
– Correlate directions also infer that vitamin levels of folate are low in relationship to high HCY [SI = 84], and B12 [SI = 45] and that high HCY levels correlate with low B6 levels [low B6 ROC in relationship to HCY [SI = 14]].
– There is no inference in the correlates to suggest that vitamin D levels are low unless there is a high zinc level, which is not a general finding associated with correlates for elevated free copper.

As judged by HPL significance levels, when the MTHFR enzyme is homozygously coded (677TT), oxidative stress is nowhere near as severe as for the folate/FAD depleted wild-type enzyme. Furthermore, catecholamines are less elevated in the 677TT genotype. Elevated Cu/Zn ratios, though only marginally related to psychosis caseness in the 677TT genotype, nevertheless play a significant role in relationship to functional disability and severity of illness. This effect is probably due to coppers inhibition of CBS, promoting HCY elevation and halting glutathione synthesis (57). Unbound copper is a powerful oxidant causing inflammation and free radical damage to the tissues (101) so its relationship to caseness is not surprising.

For this reason correlative findings for elevated free Cu/Zn ratio were examined again in the whole sample data set, with the following findings:

– High% free copper/zinc ratio ROC (*n* = 133, AUC = 0.611 *P* = 0.022), but
– free% cu/zn ratio correlates most closely with activated vitamin B6 levels (*n* = 129, rho = 0.171, *P* = 0.053), and there is no demonstrated correlative relationship to levels of folate or other vitamins (Table 2).
– as expected from the dynamics of ceruloplasmin and zinc binding with copper, an interesting marginal inverse SEM covariance was noted between ceruloplasmin (Cp) and caseness (*n* = 134, *z* = −1.87, *P* = 0.062) and % free Cu/zinc ratio is equally inversely covariant with ceruloplasmin (Cp) (*n* = 134, *z* = −3.58, *P* = 0.000) and with plasma zinc (*n* = 134, *z* = −3.37, *P* = 0.001).

High% free Cu:Zinc ratio was a sufficiently significant finding in the overall data set to meet ROC criteria for biomarker status. The fact that copper is an inhibitor of the CBS enzyme that leads to glutathione synthesis (57, 101) and vitamin B6 is a cofactor for four enzymes related to this pathway, may explain why vitamin B6 is underutilized in the setting of high free copper and why HCY is also elevated in this setting. In the total sample data set and in the SEM findings, the relationship of correlates between % free copper, zinc, and ceruloplasmin nicely fitted the known relationship that exists between excess free copper and low zinc and/or low ceruloplasmin levels (102). In an inflammatory setting, Cp is an acute phase reactant that can act as a diamine oxidase (ceruloplasminase) to enhance histamine metabolism and lower histamine levels (103). Elevated free copper, as found in this study, is also expected to support the activity of the copper containing enzyme diamine oxide (DAO), which also metabolizes histamine leading to lowered histamine levels (65, 104). In this regard, an interesting marginally significant negative relationship was found to exist between free Cu/Zn and histamine and (*n* = 134, rho = −1.95, *P* = 0.052) and this was confirmed by a marginal negative coefficient between these two variables on SEM analysis (*n* = 134, *z* = −1.95, *P* = 0.052). In this setting, NA/MHMA and AD/MHMA levels related inversely to low histamine (designated as <0.35 µg/l) (*n* = 130, rho = −0.199, *P* = 0.023), meaning that MHMA/NA and MHMA/AD (representative of good MAO catecholamine metabolism) positively correlate with low histamine levels. If this is so, that good MAO metabolism (facilitated by FAD) is ongoing in the homozygous MTHFR polymorphism setting, this lends weight to the concept that FAD may be conserved and readily available for MAO activity when the MTHFR enzyme is inactive. It explains why no correlative relationship is found between elevated NA/MHMA and AD/MHMA ROCs and the homozygous 677TT MTHFR polymorphism in the full sample set and why catecholamine entities as a whole hold no significant correlates with the polymorphism within the split sample set.

Since FAD is reported to be more easily dislodged from the structure of the MTHFR 677TT coded enzyme in its thermo-labile, low-activity form (105), one evolutionary benefit of this homozygous polymorphism may be its provision of dislodged un-utilized FAD for sustaining MAO enzyme activity and keeping vitamin B6 and D activated. This could be a useful gene adaptive strategy in famine environments devoid of meat and yellow vegetables such as previously linked to famine and the schizophrenia condition (106). This strategy may further provide some hedging against oxidative stress, which is another recognized feature of schizophrenia (107).

### MTHFR Homozygous Polymorphism, Elevated B12 Levels and Risk of Homocysteine-Driven BHMT Hyperactivity and an Over-Methylation State

In relationship to vitamin B6 and folate, HCY a significant correlate within the MTHFR homozygous data set and findings from two other studies have identified that the relationship between riboflavin and HCY is dependent on the MTHFR C677 homozygous polymorphism (108). In the split data set for the MTHFR 667TT polymorphism, vitamin levels of folate were found to be low in relationship to high HCY (Significance index 84), and B12 (Significance index 45), high-lighting the potential of this homozygous polymorphism to contribute to HCY-related cardiovascular disease. When HCY is elevated and its usual metabolism route *via* MS is inoperative due to insufficient 5MTHF from the inactive, MTHFR 677TT coded enzyme, its metabolism must proceed by the betaine [trimethyl glycine (TMG)] degradation pathway, served by the BHMT enzyme (94). This activity produces DMG that requires FAD to de-methylate itself. Since lack of correlates for catecholamines in the split sample infers that MAO activity in good in the MTHFR 677TT genotype setting, this also implies that there is sufficient FAD cofactor available for other enzyme reactions. In particular, remethylation of DMG can now proceed and with it the betaine degradation pathway, whereby elevated HCY can drive BHMT to produce high levels of SAMe from Methionine. High levels of SAMe will in turn drive catecholamine COMT metabolism (Figure 4) Therefore, in such a flavin-sufficient, methylating state, there is a theoretical risk that catecholamines will be excessively degraded by both MAO and COMT to a degree that depleted catecholamine levels lead to depression and adrenal re-synthesis exhaustion with cognitive and motor retardation (17, 109). In addition high free copper levels disturb thyroid function, adrenal hormone production and lower DA and histamine levels whilst SAMe-driven histamine metabolism also promotes low histamine levels – effects that all promote apathy and negative symptoms in schizophrenia.

### Summary of Findings and Further Implications

Taken together, findings from this study implicate the existence of two contrasting forms of functional psychosis, the differing characteristics of which are summarized in Table 9. One form associated with low flavin and folate availability with a normal MTHFR enzyme polymorphism and the other form associated with a homozygous MTHFR 677TT polymorphism in the presence of elevated free copper. The two forms may be evolutionarily linked, as the homozygous MTHFR 677TT polymorphism may be a gene adaptation designed to conserve body flavin in the setting of famine conditions (110). Other researchers have also come to a similar, though non-specific conclusion that the 677TT MTHFR polymorphism may protect against tissue injury or unbalanced vitamin intake (111).

Though these findings highlight Flavin deficiency, they broadly imply that that, there is scope for targeted, sequenced nutritional support in functional psychoses such as schizophrenia and schizoaffective disorder. Several studies have indicated widespread riboflavin (vitamin B2) deficiency in psychiatric patients as well as in youth and elderly in industrialized countries with an imputed role for flavin supplementation (112). In particular, early zinc, copper and riboflavin level assessments, vitamin level assessments and gut vitamin absorption assessments with mucosal rehabilitation could be considered primary initiatives to re-establish good vitamin absorption, in functional psychosis treatment. In the non-polymorphism MTHFR677C → T gene-variant guided setting, once reasonable diet and mucosal function has been restored, riboflavin supplementation and if necessary supplementation of other vitamins (methylfolate, and vitamins B6, and D) and zinc can be introduced. Dietary riboflavin sources should also be considered. These include almonds, whole grains, wheat germ, wild rice, mushrooms, soybeans, brewer’s yeast, milk, yogurt, eggs, broccoli, Brussels sprouts, and spinach. Riboflavin supplementation has been demonstrated to restore glutathione activity (61) and *N* acetyl cysteine, an intermediate substrate in the transulfuration pathway from homocysteine to glutathione (Figure 1), may also offset glutathione synthesis deficiency in settings of oxidative stress (113). Such combination therapy has already been proposed with zinc and B6 supplementation trialed to reduce elevated HPL levels (114). Zinc supplementation reduces excess free copper and moderates copper effects in relationship to neurotoxicity, oxidative stress, thyroid damage and poor FMN synthesis (115, 116). The relationship of low ceruloplasmin to high free copper levels and Wilson’s disease is also well-known and are deserving of greater attention in psychosis research (117). Biochemically targeted, longitudinal therapeutic trials are also required in this propitious field of precision biochemical remediation for schizophrenia and schizoaffective psychosis.

Findings from the MTHFR heterozygous data-set have not been discussed in detail in this manuscript due to the fact that correlates within this third data-set generally reflected findings in the no-polymorphism state, with some overlaps with the homozygous state findings. This is may be because the 677CT MTHFR adaptation is attempting to modify the extreme biochemical effects of either under- or over-methylation. Furthermore, the fact that three different forms of gene code for the MTHFR enzyme at position 677 and that flow-on biochemical effects of two of these (wild-type, and homozygous) form contrasting biochemical phenotypes within a MTHFR 677C → T split data-set, may explain why contrasting results are often obtained from psychiatric research where different biochemical effects of MTHFR 677C-T phenotypes in populations under examination are combining to obscure research outcomes. These findings therefore imply the need to take these MTHFR677 C → T biochemical phenotypes well into account in research data examination and outcome comparison.

### Limitations

This study used multiple exclusion criteria to obtain a highly characterized group of case and control participants. Limitations of this study have been outlined in previous publications relating to findings from the Mental Health Biomarker Project (1, 2) and relate to issues of bias inherent in a case–control study, smoking, and medication. With respect to confounding effects from medications received by only a few case participants in this study, oral contraceptives have been noted to lower vitamin B6 levels and sodium valproate noted to lower folic acid levels and raise HCY levels (118). Interestingly, some tricyclic antidepressants and antipsychotics such as chlorpromazine have been reported to inhibit synthesis of FAD from riboflavin, by the flavokinase enzyme (119). Though no patients in this study were on such antidepressants, research on riboflavin-related enzyme dynamics are certainly now warranted for a wider range of antipsychotics.

During SEM data analysis, 66 parameters were estimated from a covariance matrix of 17 independent variables and 153 data points were derived from 134 observations, so data available for estimation of SEM model parameters was minimal. Although significant correlates were discovered within the split data set for MTHFR 677TT, the overall sample number was low relative to the other part of the data set due to the lesser incidence of this polymorphism. Findings from the MTHFR heterozygous data-set have not been discussed in detail in this manuscript due to the fact that correlates within this data-set occupied an overlapping position between the other two data sets. Unknown thyroid hormone status of individuals in this study is a further limiting factor of potential relevance since diminished FMN–FAD effect hepatic flavoprotein enzyme activity which may relate to hypothyroidism and unavailability of thyroid hormone for priming FMN synthesis (120). This study’s finding that MTHFR 677TT coding acts as a threshold for two flavin-related phenotypes does not exclude the possibility of a number of other gene variants co-acting with the MTHFR 677 gene to effect threshold risk factors for psychosis.

## Summary and Conclusion

Dynamic biochemistry understandings derived from three types of data analysis were aligned in this study and provided support for the existence of two genotype-directed biochemical phenotypes for functional psychosis. One phenotype is linked with low folate and flavin availability associated with wild-type gene coding of the MTHFR enzyme. The other enzyme form is coded by a homozygous MTHFR 677TT gene-polymorphism, linked to elevated free copper and HCY. These two different flavin-related biochemical phenotypes have correlative findings suggesting that MTHFR 677CT status has potential to influence biochemistry in two opposing directions – under-methylation and over-methylation.

In the first no-polymorphism phenotype, low folate intake or low FAD availability with normal MTHFR activity, lack of folate-substrate and FAD-cofactor for the MTHFR enzyme, retards both folate and methionine cycles creating an under-methylation setting. In the methionine cycle FAD cofactor deficiency also retards cobalamin reactivation, which retards HCY conversion to methionine. FMN–FAD deprivation also retards MAO activity, leading to catecholamine elevation. Vitamin D, B6, and glutathione also lack flavin activation, creating an environment of vitamin inactivity and oxidative stress.

In the second homozygous phenotype, the relatively inactive MTHFR 677TT polymorphism seeks to prevent this scenario by allowing its enzyme-unutilized FAD cofactor to sufficiently activate vitamin D, B6, cobalamin and glutathione. However, despite this activation facility, it can still not provide sufficient methylation product to run the methylation cycle. In this setting, HCY is elevated and an alternative, FAD-dependent channel is opened up across the methylation cycle to allow high, trapped levels of HCY to be metabolized. Hyper-activation of this HCY-driven pathway results in excessive SAMe production with attendant risk of low catecholamines, low histamine and ultimately adrenal exhaustion.

These understandings increase knowledge of the genetic and biochemical nature of schizophrenia and schizoaffective psychosis. The two contrasting phenotypes provide a structural basis for data-interpretation in future Psychiatry research and also point toward MTHFR 677 C → T gene-guided therapy as part of rational, biochemical treatment solutions in the clinic.

## Ethics Statement

Ethics permission for the study was obtained from the Queen Elizabeth Hospital Research Ethics Committee (No: 2009139) and all protocols and methods used in the project conformed to that committee’s relevant regulatory standards.

## Author Contributions

The author of this paper: SF-W, MBBS, BSc (Biochem/Pharmacol), FRANZCP (Child and Adolescent Psychiatrist), is an Honorary, visiting research fellow of the University of Adelaide (2010 onwards). As chief researcher, SF-W conceived the study concepts and initiated the study, planned the study, obtained ethics permission, designed and drove the project and selected all candidate markers, carried out data collection and sensory assessments, entered initial data, specified all data conversions, specified and guided directions of statistical inquiry, interpreted results of the data, requested ancillary data and analyses as required, drafted, prepared, and wrote all versions of this manuscript, including formatting tables and placing all data in them and designing diagrams. Beginning in 2012, SF-W directed phases of data analysis and made specific requests for data analysis towards finding an integrated theory and requested data in relationship to MTHFR polymorphism activity and the correlation of variables for homocysteine, histamine, elevated Noradrenaline, Adrenaline and Dopamine, % free copper, ceruloplasmin, zinc, vitamin B6, vitamin D, vitamin B12, monoamine oxide activity (NA/MHMA and AD/MHMA), and FMN/FAD availability. SF-W also requested confirmation of Flavin role by pathway analysis and discovered the different patterns of biochemical phenotypes between MTHFR 677C → T-no polymorphism status (under-methylation) and MTHFR homozygous polymorphism (over-methylation) within schizophrenia and schizoaffective disorder. Also the no-polymorphism and homozygous MTHFR relationships to the heterozygous gene form and to other biochemical variables and their ROCs with respect to psychosis caseness, severity, disability, and other functional parameters.

## Conflict of Interest Statement

The Author reports no conflict of interest at the time of undertaking this research or writing this paper. A provisional patent application was filed in September 2016.

## Abbreviations

AD, adrenaline; BHMT, betain-homocysteine *S*-methyltransferase; Ca+, +Calcium; CBS, cystathione beta synthase; COMT, catechol-*o*-methyltransferase; Cp, ceruloplasmin; DA, dopamine; DAO, diamine oxidase; DOI, duration of illness; FAD, flavin dinucleotide; FMN, flavin mononucleotide; GSH, reduced (active) glutathione; GSI, Global Severity Index; GSSH, oxidized form of glutathione; HCY, homocysteine; HPL, hydroxypyrroline-2-one; MAO, monoamine oxidase; MHMA, methylhydroxymandelic acid; MS, methionine synthas; MTHF, 5-methyl tetrahydrofolic acid (active form of folate) produced by the MTHFR enzyme; MTHFR, 5,10-methylenetetrahydrofolate reductase enzyme; NA, Noradrenaline; SAMe, *S*-adenosyl homocysteine; SIR, symptom intensity rating; SOFAS, social and occupational functioning assessment scale; Vitamin B2, riboflavin.
