# Supplementary material for: Fundamental Role of Methylenetetrahydrofolate Reductase 677 C → T Genotype and Flavin Compounds in Biochemical Phenotypes for Schizophrenia and Schizoaffective Psychosis
Source: Front Psychiatry. 2016 Nov 9;7:172. doi: 10.3389/fpsyt.2016.00172 (PMC5102045; doi:10.3389/fpsyt.2016.00172)
Supplement: Supplementary file 1 [file Presentation_1.pdf]

Supplementary Material

Fundamental role of Methylenetetrahydrofolate Reductase 677 C->T genotype and Flavin compounds in biochemical phenotypes for schizophrenia and schizoaffective psychosis.

Stephanie Fryar-Williams[1,2,3]

[1]Youth in Mind Research Institute, Norwood, SA, Australia.

[2]The Queen Elizabeth Hospital, Woodville, SA, Australia.

[3]Basil Hetzel Institute for Translational Health Research, Woodville, SA.

*** Correspondence:** Stephanie Fryar-Williams MBBS BSc. FRANZCP.
Youth in Mind Research Institute 106 The Parade, Norwood, South Australia, 5067. Phone +61 411310449. E mail sfwilliams@senet.com.au

Supplementary Sections S1-S13.

S1 Pilot Study Summary
S2 Exclusion criteria
S3. Severity and disability outcome measures, with citations.
S4. Methods for candidate biochemical markers, including equipment and citations.
S5. Further methods for candidate biochemical markers, including equipment and citations
S6. Summary of biomarker results.
S7. Sample characteristics for recruitment outcomes, medications, data imputation.
S8. Compound biomarkers of further theoretical interest

S1 Pilot study summary

A case series pilot study was conducted in 2007-2009. In this study, 10 out of 15 cases with schizophrenia (67%, P 0.002) and 7 out of 8 cases with auditory processing disorder (87%, P0.0002) had accumulated three or more abnormal biochemistry markers, when compared with 15 in-clinic selected control cases. These abnormal markers were for homocysteine, hydroxyhaempyrroline-2-one (HPL), folate, zinc, percent free copper and histamine. From these need for a minimum number of 60 cases and 60 matched controls, was estimated for 95% confidence interval power for a phase II case-control study.

S2 Exclusion criteria

Exclusion criteria included medication with Clozapine, Olanzapine which are frequently-prescribed medications for ward and outpatient clinic patients with repeated admissions for psychosis. Together with antihistamines, the following medications have prominent histamine- binding effects, so were excluded as histamine was a candidate biomarker. Patients taking antipsychotic-agents such as Zuclopenthixol, Modecate, Amisulpride, Quetiapine and Risperidone were included. Persons on mood stabilizing medications were allowed. Persons with active or unremitted use of alcohol or other substance abuse were excluded, since this can confound neurotransmitter results. Persons with organic cerebral damage as evidenced by a clinically documented, investigated or descriptive history of hospitalized head injury, unconsciousness or central nervous system disease were also excluded**,** as were persons with upper respiratory tract infections, middle ear congestion or known sensory or

learning disability. Persons with extra-pyramidal signs in ocular, arm or hand muscles were excluded prior to consent. Persons receiving vitamin therapy were also excluded due to the inclusion of vitamins as candidate markers. It was not possible to exclude smoking and have any chance of patient recruitment. Persons taking anti-histamine medication or vitamin supplementation similar to candidate markers, were also xcluded.

S3. Severity and disability outcome measures, with citations.

Functional Rating Scale	Citation
The Brief Psychiatric Rating Scale (BPRS)	Overall JE and Gorham DR (1962) The Brief Psychiatric Rating Scale.
Positive and Negative Syndrome Scale for schizophrenia (PANSS)	Kay SR, Fiszbein A and Opler LA (1987) The positive and negative syndrome scale (PANSS) for schizophrenia. *Schizophrenia Bulletin* 13(2): 261-276.
Clinical Global Impression of Severity (CGI)	Guy W (1976) Clinical global impressions [CGI]. *ECDEU assessment manual for psychopharmacology*. National Institute of Mental Health, Early Clinical Drug Evaluation, Psychopharmacology Research Branch, Rev. Rockville, MD, U.S.
Global assessment of Function (GAF)	Frances A, Incus HA and First MB (1994) *Diagnostic and Statistical Manual of Mental Disorders*, 4th edition, American Psychiatric Association, Washington, DC.
Social and Occupational Functioning Assessment Scale (SOFAS)	Goldman HH, Skodol AE and Lave TR (1992) Revising axis V for DSM-IV: a review of measures of social functioning. *American Journal Psychiatry*

S4. Methods for candidate biochemical markers, including equipment and citations.

Nutrition-Biochemistry	All fasting blood samples, collected between 9 and 11 am daily. By arrangement, transported directly to the laboratory with no storage.	Laboratory/Reference
Vitamin D (25-OH)	Diasorin Liason assay kit, for use on the Liaison platform. (nmol/L)	Clinpath Laboratories, 19 Fullarton Rd, Kent Town. South Australia 5067<br>+61 8 8366 2000.
Serum total Vitamin B12	Competitive Electrochemiluminescent Immunoassay. Roche Modular E 170<br>Automated Immunoassay Analyser, using Roche Vitamin B12 Reagent. (nmol/L)	Clinpath Laboratories. As above.
Plasma Red Cell Folate	Competitive Electrochemiluminescent Protein Binding Assay, using Roche Modular E 170, using Roche Folate Red Blood Cell (RBC) Reagent and Roche Folate RBC Haemolysing Reagent on Automated Immunoassay Analyser. (nmol/L)	Clinpath Laboratories. As above.
Serum Vitamin B6 (Pyridoxal- 5'-phosphate coenzyme form)	Whole blood High Pressure Liquid Chromatography with fluorescent detection. Chromsystems Vitamin B6 in Whole Blood High pressure Liqid Chromatography Reagent Kit. Waters Alliance 2695 Separations Module. Waters 474 Fluorescence Detector.(nmol/L)	Sullivan Nicolaides Pathology<br>143 Whitmore St, Taringa. Queensland 4068. Australia. +61 7 337 8666

Serum Copper	Flame Atomic Absorption Spectrophotometry. Varian AA-240FS. (umol/L)	Douglass Hanly Moir Pathology 14 Griffnock Avenue, Macquarie Park. New South Wales 2113. +61 2 9855 5222.
Plasma Red Cell Zinc	Inductively coupled plasma mass spectroscopy	Sullivan Nicolaides Pathology.
Serum Ceruloplasmin	Immunoturbidimetric method, using 6K91-30	Douglass Hanly Moir Pathology.
Percentage Free Copper/Red Cell Zinc	Percentage of free copper in the serum calculated by an equation based on the molecular and atomic weights of ceruloplasmin and copper (one ceruloplasmin molecule binds to six copper atoms). The ratio of the percentage free copper to red cell zinc was calculated as "percentage free copper" / "Red cell zinc umol/L".	Calculated by authors
**Intermediate substrates and enzymes**		
MTHFR Ala222Val (C677T) methyl tetrahydrofolate reductase polymorphism	Real time PCR analysis Roche Diagnostics Light-Cycler 480 kit. Using TecnoBiol reagents, Sigma probes and primers on Roche LC480 analyser.	Douglass Hanly Moir Pathology.
Plasma homocysteine	Ice transported EDTA sample. Competitive Chemiluminescent Immunoassay, using Seimens Homocysteine reagent on Seimens Advia centaur Automated Immunoassay.(umol/L)	SA Pathology. +61 8 8222 3000
Serum histamine	Beckman Coulter Radio Immunoassay, using Beckman Coulter R.I.A. Kit on Perkin Elmer Wizard 1470 Automated Gamma Counter. (umol/L)	Sullivan and Nicolaides.

S 5. Further methods for biochemistry markers, including equipment and citations.

Test	Method, Analyzer, Reagents.	Laboratory/Reference
**Neurotransmitters**		
Biogenic amines: Dopamine, Noradrenaline and Adrenaline,	Spot-baseline (fasting) urinary neurotransmitter testing (second void morning), snap-frozen to minus 30 degrees and analysed by mass spectrometry, using nanomols per millimol of urinary creatinine as a standard.	SA Pathology, Adelaide, South Australia. Whiting MJ. 2009. Simultaneous measurement of urine metanephrines and catecholamines by liquid chromatography with tandem mass spectrometric detection. *Annals of Clinical Biochemistry*, **46**:129-136
Creatinine	Spot urine specimen from the same void as biogenic amines, expressed in (millimols per Litre)	SA Pathology, Adelaide SA.
**Oxidative stress: Urinary hydroxyhemopyrro**	**Fasting urine sample collected whilst patient at rest, separated from blood drawing by minimum of 2 hours.**	

S6. Summary of single biomarker results (XLSTAT, alpha 0.05).

	AUC	p-value (Two-tailed)	Sensitivity	Specificity	PPV	NPV	Accuracy
**DA (n 133)**	0.687	< 0.0001	0.364	0.94	0.058	0.993	0.661
**NA (n 133)**	0.835	< 0.0001	0.792	0.791	0.037	0.997	0.792
**Adrenaline (n 133)**	0.836	< 0.0001	0.74	0.821	0.04	0.997	0.778
**Free Cu:Zn (n 133)**	0.599	0.032	0.468	0.761	0.019	0.993	0.603
**Vitamin B6 (n 129)**	0.647	0.001	0.808	0.484	0.016	0.996	0.662
**RC Folate (n 133)**	0.659	0.000	0.577	0.731	0.021	0.994	0.648
**Vit D (n132)**	0.664	0.000	0.61	0.687	0.019	0.994	0.646
**HPL/Creatinine (n 133)**	0.696	<0.0001	0.697	0.642	0.009	0.998	0.669

S7. Sample characteristics: for catchment area, recruitment outcomes, medications, data imputation. S6. Sample characteristics: for catchment area, recruitment outcomes, medications, data imputation.

The case sample:

- were diagnosed with schizophrenia and schizoaffective disorder
- drawn from a sample pool of 22,000.
- 4 eligible cases of psychosis were allocated a diagnosis of psychosis for investigation
- Imposition of multiple exclusion criteria restricted eligible cases to 370
- only twenty five percent of these consented.
- This high refusal rate resulted in 89 consenting cases,
- 7 did not reach assessment due to declining mental state and
- 15 were excluded due to the confounding factor of SSRI, SNRI medication.
- number of cases in the final analysis was 67.

The control sample:

- was drawn from an available sample number of 2489.
- were matched for age and sex with patients.
- randomization and imposition of recruitment exclusion criteria
- after a low consent rate of 25 per cent, a total of 72 control participants were recruited.
- 5 of these were excluded due to failure to meet exclusion criteria on assessment.
- no control had a diagnosis of schizophrenia or any DSM-diagnosed mental illness,
- all control participants were rated before biological assessment for reported and observed subclinical symptoms in a real-world setting, by a psychiatric trained assessor who was not blind to their study status.
-

		Schizophrenia		Schizoaffective psychosis		Psychosis FI		Cases		Controls		Total Participants	
		n	%	n	%	n	%	n	%	n	%	n	%
Sex	Female	12	36.4%	16	53.3%	2	50.0%	30	44.8%	34	50.7%	64	47.8%
	Male	21	63.6%	14	46.7%	2	50.0%	37	55.2%	33	49.3%	70	52.2%
Persons		33	100.0%	30	100.0%	4	100.0%	67	100.0%	67	100.0%	134	100.0%

Sample characteristics (SE = standard error)

Character-istics	Schizophrenia			Schizoaffective Psychosis			Psychosis FI			Cases			Controls			Total Participants		
	n	Mean	SE	n	Mean	SE	n	Mean	SE	n	Mean	SE	n	Mean	SE	n	Mean	SE
**Age**	33	40.7	2.0	30	40.8	1.9	4	37.3	7.4	67	40.5	1.3	67	45.7	1.4	134	43.1	1.0
**Age of onset**	31	23.9	2.0	27	22.5	1.5	3	32.7	10.1	61	23.7	1.3	0			61	23.7	1.3
**Duration of Illness (DOI)**	31	16.5	1.9	27	18.0	2.3	3	8.0	1.0	61	16.8	1.4	0			61	16.8	1.4
**Symptom Intensity Rating (SIR)**	33	113.8	5.7	30	94.4	5.7	4	119.5	14.8	67	105.4	4.0	67	42.8	.3	134	74.1	3.4
**Body Mass Index (BMI)**	23	31.1	2.3	27	29.6	1.4	3	25.8	1.2	53	30.0	1.2	66	26.7	.6	119	28.2	.6
**Right hand dominance %**	32	92.1	3.0	29	94.5	2.4	4	82.5	17.5	65	92.6	2.1	67	93.1	1.7	132	92.8	1.3
**Urine Creatinine (mmol/L)**	33	8.8	.9	29	9.3	1.2	4	12.3	5.1	66	9.2	.7	67	9.5	.7	133	9.3	.5
**5 - Hydroxyl indole acetic acid (5-HIAA)**	33	4.6	1.4	29	3.3	.8	4	9.5	4.8	66	4.3	.8	67	1.6	.1	133	2.9	.4
**Plasma homocysteine (umol/L)**	33	10.5	.4	29	9.5	.5	4	9.3	1.4	66	10.0	.3	66	9.5	.3	132	9.7	.2
**Red cell acetylcholine esterase (U/gb Hb)**	29	38.3	.9	29	41.6	1.2	3	37.3	1.9	61	39.8	.7	67	39.6	.7	128	39.7	.5
**Hearing Threshold (Db)**	31	548.4	27.0	23	587.0	67.8	3	500.0	0.0	57	561.4	30.8	60	550.0	19.5	117	555.6	18.0
**Visual threshold of near vision**	32	6.5	.6	26	6.0	.7	3	8.0	3.0	61	6.3	.5	67	5.2	.1	128	5.7	.2

(STATA analysis)	Schizophrenia	. Condition Schizoaffective	Other	Total
ABILIFY	1	1	0	2
AMISULPRIDE	1	1	0	2
CHLORPROMAZINE	1	1	0	2
FLUPENTHIXOL	1	1	0	2
HALOPERIDOL	0	1	0	1
LITHIUM	0	3	0	3
BENZODIAZEPINE	2	2	0	4
PALIPERIDONE	1	0	0	1
QUETIAPINE	2	8	0	10
RISPERIDONE	18	7	3	28
VALPROATE	2	2	0	4
ZIPRAZIDONE	1	1	0	2
ZUCLOPENTHIXOL	4	1	0	5
MODECATE	0	1	0	1
OTHER	1	9	1	11
Total	35	39	4	78
Cases	29	28	4	61
Valid cases:	61			
Missing cases:	6			

Domain type	Observed	Missing	Total	% data missing
Laboratory-derived variables	126	2	134	1.5
Visual variables	120	11	134	8.2
Auditory variables	120	10	134	7.5

S8. Compound biomarkers of further theoretical interest.

	XLSTAT				STATA			
	**n**	**AUC**	**SE**	**p**	**n**	**AUC**	**SE**	**p**
MTHFR polymorphismn n=69	134	0.5109	0.000	.	134	0.5109	0.0445	0.4032
**MTHFR homozygous polymorphism n=7**	134	0.5377	0.000		134	0.5377	0.1034	0.3577
NA_MHMA	133	0.7915	0.0386	< 0.0001	133	0.7915	0.0402	0.0000
HIAA	133	0.6774	0.0000	.	133	0.6774	0.0427	0.0000
Histamine	134	0.5755	0.0000	.	134	0.5755	0.0493	0.0628
Histamine NA	134	0.8389	0.0355	< 0.0001	134	0.8389	0.0363	0.0000
Plasma HCY	132	0.5574	0.0491	0.1213	132	0.5574	0.0502	0.1264
Serum B12	134	0.5650	0.0491	0.0928	134	0.5650	0.0497	0.0955
MHMA	133	0.6776	0.0000	.	133	0.6776	0.0420	0.0000
AD_MHMA	130	0.7865	0.0000	.<0.0001	130	0.7865	0.0402	0.0000
DA/HVA	133	0.5828	0.0488	0.0450	133	0.5828	0.0494	0.0469
